# Sirolimus treatment for intractable vascular anomalies (SIVA): An open‐label, single‐arm, multicenter, prospective trial

**DOI:** 10.1111/ped.70002

**Published:** 2025-03-26

**Authors:** Michio Ozeki, Saori Endo, Shiho Yasue, Ryuta Asada, Akiko M. Saito, Hiroya Hashimoto, Shigeru Ueno, Shoji Watanabe, Motoi Kato, Kyoichi Deie, Shunsuke Nosaka, Mikiko Miyasaka, Akihiro Umezawa, Kentaro Matsuoka, Mototoshi Kato, Tatsuo Kuroda, Takanobu Maekawa, Satoshi Hirakawa, Taizo Furukawa, Shigehisa Fumino, Tatsuro Tajiri, Junkichi Takemoto, Naonori Kawakubo, Akihiro Fujino

**Affiliations:** ^1^ Department of Pediatrics, Graduate School of Medicine Gifu University Gifu Japan; ^2^ Clinical Research Center NHO Nagoya Medical Center Nagoya Japan; ^3^ Innovative and Clinical Research Promotion Center, Graduate School of Medicine Gifu University Gifu Japan; ^4^ Core Laboratory Nagoya City University Graduate School of Medical Sciences Nagoya Japan; ^5^ Department of Pediatric Surgery Tokai University School of Medicine Hiratsuka Japan; ^6^ Department of Plastic Surgery Saitama Children's Medical Center Saitama Japan; ^7^ Department of Plastic and Reconstructive Surgery Okayama University Hospital Okayama Japan; ^8^ Department of Pediatric Surgery Kitasato University School of Medicine Sagamihara Japan; ^9^ Department of Radiology National Center for Child Health and Development Tokyo Japan; ^10^ National Center for Child Health and Development Research Institute Tokyo Japan; ^11^ Department of Pathology Tokyo Metropolitan Children's Medical Center Tokyo Japan; ^12^ Department of Pediatric Surgery Keio University School of Medicine Tokyo Japan; ^13^ Department of General Pediatrics & Interdisciplinary Medicine National Center for Child Health and Development Tokyo Japan; ^14^ Department of Dermatology Hamamatsu University School of Medicine Shizuoka Japan; ^15^ Department of Pediatric Surgery Kyoto Prefectural University of Medicine Kyoto Japan; ^16^ Department of Pediatric Surgery, Graduate School of Medical Sciences Kyushu University Fukuoka Japan; ^17^ Division of Surgery, Department of Surgical Subspecialties National Center for Child Health and Development Tokyo Japan

**Keywords:** mammalian target of rapamycin, sirolimus granule, vascular malformations, vascular tumors, venous malformations

## Abstract

**Background:**

Intractable vascular anomalies (VAs), including vascular tumors and venous, lymphatic, and mixed malformations, often have severe symptoms and a poor prognosis, highlighting the need for new treatments. We conducted a prospective trial of sirolimus (tablet and granule forms) for the treatment of VAs.

**Methods:**

In this open‐label, single‐arm, multicenter trial across four Japanese institutions, patients with VAs received oral sirolimus daily, targeting a trough concentration of 5–15 ng/mL. We evaluated response rates (radiological volume changes in lesions), skin lesions, performance status, respiratory function, visceral symptoms (bleeding, pain), laboratory data, quality of life, and safety at 12, 24, and 52 weeks.

**Results:**

Thirteen patients with VAs were treated with sirolimus. Seven patients (53.8%; 95% confidence interval: 25.1%–80.8%) showed a partial radiological response at 24 weeks, with no complete responses, and 61.5% had a partial response by 12 weeks, with little subsequent change in patients who had stable disease thereafter. Improvements in skin lesions, blood coagulation, and activities of daily living were noted. Common adverse events included stomatitis, dermatitis, diarrhea, and fever.

**Conclusions:**

Sirolimus may reduce VA tissue volume and potentially improve symptoms and activities of daily living in patients with VAs.

## INTRODUCTION

Vascular tumors and malformations, collectively known as vascular anomalies (VAs), primarily manifest in childhood and involve the abnormal development of blood and lymphatic vessels throughout the body.^1^ VAs can lead to a range of symptoms, including unilateral limb overgrowth, pain, ulcers, functional limitations, and organ complications, presenting significant management challenges. Clinical trials have targeted various intractable VAs, including kaposiform hemangioendothelioma (KHE), tufted angioma (TA), cystic lymphatic malformation (LM), generalized lymphatic anomaly (GLA), Gorham–Stout disease, venous malformation (VM), blue rubber bleb nevus syndrome (BRBNS), mixed vascular malformation, and Klippel–Trenaunay syndrome (KTS).

There is currently no standard treatment for these intractable vascular tumors and malformations, and no medications with proven efficacy have been approved for these conditions.^1^ Current treatments, such as steroids, interferon, and propranolol, have limitations, with long‐term steroid use associated with the risk of side effects. Notably, however, recent research has highlighted the importance of the phosphoinositide 3‐kinase/protein kinase B (Akt)/mammalian target of rapamycin (mTOR) pathway in the development of blood and lymphatic tissues, prompting the development of new therapeutic drugs targeting this pathway.^2^ mTOR regulates cell division, proliferation, and survival, and sirolimus accordingly works by binding to mTOR and inhibiting its activation, leading to decreased cell proliferation, anti‐angiogenesis, and anti‐lymphangiogenesis effects, and effectively reducing mutant TIE2‐induced Akt signaling.^3^, ^4^ Sirolimus acts on endothelial cells in lesions, reducing lymph fluid production and leakage, thereby shrinking the lesions. Sirolimus may reduce pain, bleeding, lesion size, functional and esthetic impairment, and intravascular coagulopathy, thus improving various clinical symptoms.^5^

Sirolimus is an orally administered medication that is gaining attention as a new treatment for difficult‐to‐treat vascular tumors and malformations.^5^, ^6^ It was previously only available in Japan in tablet form^6^; however, Nobelpharma Co., Ltd. has developed a granule formulation, which has been pharmaceutically approved and is now available in Japan. This is a crucial advancement, particularly for patients who struggle to swallow tablets, such as infants and those receiving enteral nutrition. This study aimed to evaluate the effectiveness and safety of both forms of sirolimus for managing VAs.

## METHODS

### Study objectives, design, and enrollment

We conducted an open‐label, single‐arm, multicenter, prospective clinical trial, Sirolimus for Intractable Vascular Anomalies, at four institutions in Japan, to evaluate the effectiveness and safety of sirolimus for treating VAs. Approval for this clinical trial was obtained from the institutional review boards of each participating facility. This study has been registered at the Clinical Trial Registry (UMIN‐CTR) (UMIN000038973, https://center6.umin.ac.jp/cgi‐open‐bin/ctr_e/ctr_view.cgi?recptno=R000044448).

Patients who met the eligibility criteria and provided informed consent and assent were enrolled. Before enrollment, all patients were reviewed by a multi‐institutional, multidisciplinary evaluation committee, including individuals from pediatric surgery, pediatrics, and radiology departments. Clinical information, clinical photographs, and radiological findings were assessed. There are no globally established diagnostic criteria for VAs, and we therefore used the Japanese domestic diagnostic criteria for each disease.^1^ The inclusion criteria were: patients with a corrected age >1 month at the time of obtaining consent; confirmed diagnosis of KHE, TA, cystic LM, GLA, Gorham–Stout disease, VM, BRBNS, mixed vascular malformations, or KTS; presence of at least one target lesion (e.g., tumor, venous or lymphatic lesion) measurable on magnetic resonance imaging (MRI); and the presence of severe and intractable symptoms resulting from the target disease (including bleeding, chronic pain, recurrent cellulitis [>three episodes per year], ulceration, involvement of internal organs and/or bones, and potential impact on organ function, including eyes, airway, and ears).

The exclusion criteria were: patients with a Karnofsky performance status (PS) score ≤ 30 for those aged ≥10 years or a Lansky play‐PS score ≤ 30 for those aged <10 years; patients with impaired liver, kidney, or cardiac function; patients who had received molecularly targeted drugs affecting the mTOR pathway, immunosuppressive medications, or drugs that inhibit or induce CYP3A4 enzyme activity; and patients who had undergone surgery (resection, sclerotherapy, or endovascular treatment) or received therapeutic drugs (such as propranolol, Kampo medicines, interferon, octreotide, bisphosphonate, or denosumab), chemotherapy agents, or radiation therapy for the target lesion, due to potential impacts on the assessment of the target lesions.

### Treatment and assessments

Patients received sirolimus tablets or granules (Rapalimus Tablet or Granules, Nobelpharma Co., Ltd., Tokyo, Japan) orally once a day, either after meals or on an empty stomach. The initial dose of oral sirolimus tablets in patients weighing ≥30 kg was 2 mg/day. The starting dose of sirolimus granules in patients ≥30 kg was 0.7 g, equivalent to 1.4 mg of sirolimus, and the initial dose in patients <30 kg was calculated based on the patient's age and weight (Table 1). Dose adjustments were made according to sirolimus trough concentrations measured in the second week, to achieve a target concentration of 5–15 ng/mL. There was no specified maximum dosage limit. Trough concentrations were measured after the first and second weeks, and every 4 weeks thereafter. If a change in sirolimus formulation was requested at Week 25 of administration, the patient could switch to the other formulation, and the dose was adjusted on the basis that 1 mg of the tablet formulation was equivalent to 0.7 mg of granules. Supportive treatments, such as antibiotics, blood transfusions, antipyretics, and analgesics, were permitted if they did not interfere with assessment of the VA lesions, but additional drugs that might interact negatively with sirolimus, drugs affecting CYP3A4 enzyme activity, and treatments such as surgery, sclerotherapy, or other drugs that could influence evaluation of the target lesions were not allowed.

**TABLE 1:** Initial dose of sirolimus granules in subjects weighing >30 kg at the time of enrollment.

Age	Initial dose (mg) per day (amount of granules)
<3 months	0.02 mg/kg (0.01 g/kg)
3–6 months	0.04 mg/kg (0.02 g/kg)
6–12 months	0.06 mg/kg (0.03 g/kg)
≥12 months	0.08 mg/kg (0.04 g/kg); max dose 1.4 mg (0.7 g)

The primary outcome was the radiographically determined objective response rate to sirolimus, defined as the number of patients who achieved either a complete response (CR) or a partial response (PR), assessed by MRI evaluation of the target‐lesion volume at the end of 24 weeks. For unbiased evaluation, responses were assessed by two central radiologists, independent of the study investigators, who measured the dimensions of the venous or lymphatic lesions using fat‐saturated T2‐weighted MRI sequences, excluding other pathological changes such as inflammation, bleeding, or hematoma. The dimensions were determined using a region of interest tool within Digital Imaging and Communications in Medicine viewer software (OsiriX© v.9.0; Pixmeo, Bernex, Switzerland). The target‐lesion volume was calculated by multiplying the sum of the region of interest areas by the interslice distance. Notably, non‐lymphatic tissues, including bleeding, inflammation, or hematoma, were not considered in the volume calculations. The criteria for evaluating the MRI findings were classified as follows: CR indicated the total disappearance of all target lesions; PR was defined as a decrease in target‐lesion volume ≥ 20% compared with the baseline volume; progressive disease (PD) was defined as an increase in volume ≥ 20% compared with the smallest volume; and stable disease (SD) was defined as a volume change that did not meet the criteria for PR or PD.

The secondary outcomes included radiological, clinical, and biological evaluations of sirolimus efficacy and safety, including radiological response rate (12 and 52 weeks), patient status, complications related to internal organs (e.g., pleural effusion, ascites, bleeding, and pain), skin lesion alterations, and laboratory tests (complete blood count, liver function, lipid profile, and coagulation parameters). Quality of life (QOL) was also assessed at 12, 24, and 52 weeks, and vital signs and pharmacokinetic and safety profiles, including adverse events, were monitored every 4 weeks. Patient status, bleeding, pain, QOL, and adverse events were quantified using the Karnofsky PS for individuals aged ≥10 years, or the Lansky Play‐PS for children <10 years. The World Health Organization (WHO) Bleeding Scale,^7^ a visual analog scale for pain,^8^ the Pediatric Quality of Life Inventory (PedsQL™) 4.0 Generic Core Scales for individuals >25 years^9^ and the Functional Assessment of Cancer Therapy‐General (FACT‐G) for individuals ≥25 years,^10^ and the Common Terminology Criteria for Adverse Events version 4.0 were also employed for respective measures.^11^ Concerning skin lesions, patients with skin lesions at the initiation of sirolimus treatment were evaluated subjectively by physicians for any change in size and color, using a six‐point scale: markedly improved, improved, slightly improved, unchanged, slightly worsened, and worsened.

### Sample size and data analysis

There is a lack of data regarding the natural progression of intractable VAs, and we therefore analyzed retrospective data from a national Japanese survey on intractable VAs.^9^ In this survey of 91 patients with intractable VAs, radiological improvement sustained for ≥1 year was observed in only one patient (1.1%), establishing a response threshold of 5%. The anticipated response rate was set at 60%, based on previous clinical trials that reported a radiological response rate of 61.3% (38/62 patients).^5^ The sample size was accordingly determined based on the probability that the lower bound of the exact 95% confidence interval (CI) for the primary outcome would surpass the 5% threshold. Seven patients were required to achieve a statistical power of ≥90%. We therefore aimed for a sample size of 10 patients to allow for potential drop‐outs.

For the primary outcome, we assessed the response rate (proportion of patients with CR or PR) at 24 weeks and 95% CIs in the full analysis set, including all enrolled patients. The efficacy of sirolimus was affirmed if the lower 95% CI was >5%. Regarding the secondary outcomes, QOL scores were estimated by calculating adjusted means and CIs for change from pre‐treatment using a linear mixed‐effects model with fixed effects for time points and variable effects for patients, and pre‐treatment and post‐treatment PS scores were compared using Wilcoxon's signed rank test.

## RESULTS

**FIGURE 1:**
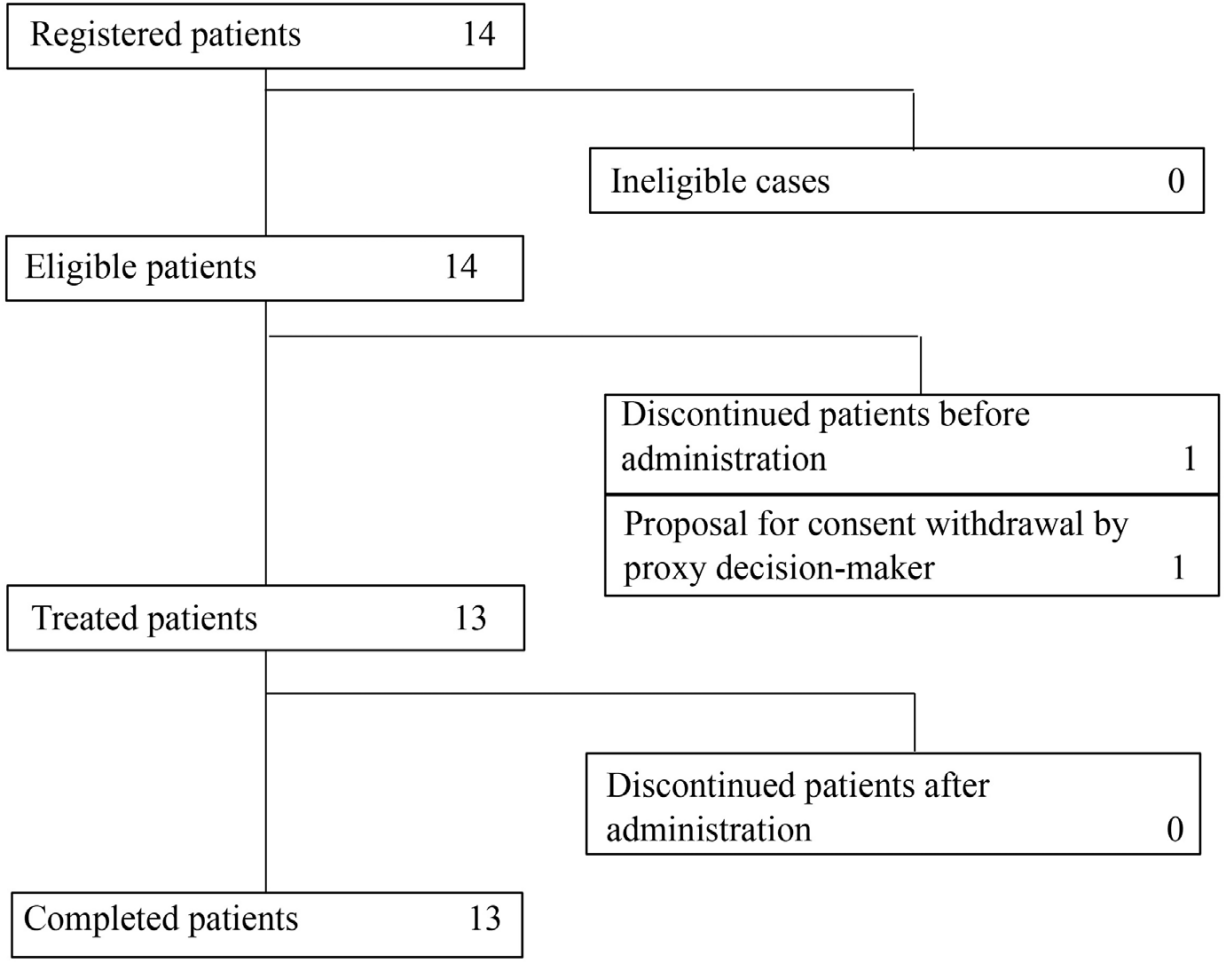
Patient distribution and inclusion in the analysis.

A total of 14 patients were enrolled in the study, of whom 1 (Case 12) was excluded due to a guardian's withdrawal of consent. Thirteen patients (three cystic LM, three KTS, two BRBNS, two mixed vascular malformations, one KHE, one GLA, and one VM) were therefore finally evaluated. No patients discontinued treatment during the course of the trial (Figure 1). At the start, the drug forms were granules in 9 of the 13 cases (69.2%) and tablets in 4 of the 13 cases (30.8%). One patient (Case 5) switched from granules to tablets at 25 weeks, and one patient (Case 1) switched from tablets to granules.

The demographic characteristics of the patients treated with sirolimus (full analysis set) are shown in Table 2. Eleven patients had some complications. Twelve patients had abnormal skin lesions before treatment. The target lesions for assessment comprised seven appendicular lesions (right shoulder, right leg, left leg, from left shoulder to forearm, knee, right leg, and left shoulder), four subcutaneous trunk lesions (right chest, left chest, axillary fossa, and right thoracoabdominal), and two cervical lesions. Bleeding from any site was observed in five patients (38.5%), including three cases (23.1%) of grade 1 and two cases (15.4%) of grade 2. The median drug‐taking rate was 100% (range: 81.9%–100%).

**TABLE 2:** Characteristics of patients treated with sirolimus (full analysis set).

Characteristic		Number (*n* = 13)
Sex (female/male)		7/6 (53.8%/46.2%)
Age at start of sirolimus (years)		6.0 ± 18.8 (0–71)^a^
	<2	4 (30.8%)
	2–11	5 (38.5%)
	12–19	3 (23.1%)
	≥20	1 (7.7%)
Height (cm)		111.0 ± 40.6 (65–181)^a^
Body weight (kg)		19.7 ± 22.2 (6.9–69.7)^a^
BMI (kg/m^2^)		16.7 ± 4.1 (14.5–28.3)^a^
BSA (m^2^)		0.78 ± 0.53 (0.36–1.87)^a^
Name of VAs	Cystic LM	3 (23.1%)
	KTS	3 (23.1%)
	BRBNS	2 (15.4%)
	Complex VAs	2 (15.4%)
	KHE	1 (7.7%)
	GLA	1 (7.7%)
	VM	1 (7.7%)
Disease duration^b^ (years)		7.0 ± 6.7 (1–19)^a^
PS	Karnofsky PS score (≥10 years old) (*n* = 4)	80.0 ± 10.0 (60–80)^a^
	Lansky play‐performance scale (<10 years old) (*n* = 9)	70.0 ± 15.0 (60–100)^a^
Complications	Anemia	3 (23.1%)
	Dry eczema	2 (15.4%)
	Others^c^	1 (7.7%)

Abbreviations: BMI, body mass index; BRBNS, blue rubber bleb nevus syndrome; BSA, body surface area; GLA, generalized lymphatic anomaly; KHE, kaposiform hemangioendothelioma; KTS, Klippel–Trenaunay syndrome; LM, lymphatic malformation; PS, performance status; VA, vascular anomaly; VM, venous malformation.

^a^ Median ± standard deviation (minimum–maximum).

^b^ Duration from diagnosis to administration of sirolimus.

^c^ Kasabach–Merritt phenomenon, atopic dermatitis, hemoglobin decrease, gastrointestinal tract insertion, enuresis, dry skin, seasonal allergies, macrodactyly, thrombocytopenia, increased lactate dehydrogenase in blood, oral herpes, dry mouth, neutropenia, decreased neutrophil count, hypertension, syndactyly, osteoporosis, eczema, hypofibrinogenemia, dysuria, leukopenia, rhinitis, obstructive airway disorder, migraine, constipation, asthma, pain, urticaria, and dental caries.

### Outcomes

**FIGURE 2:**
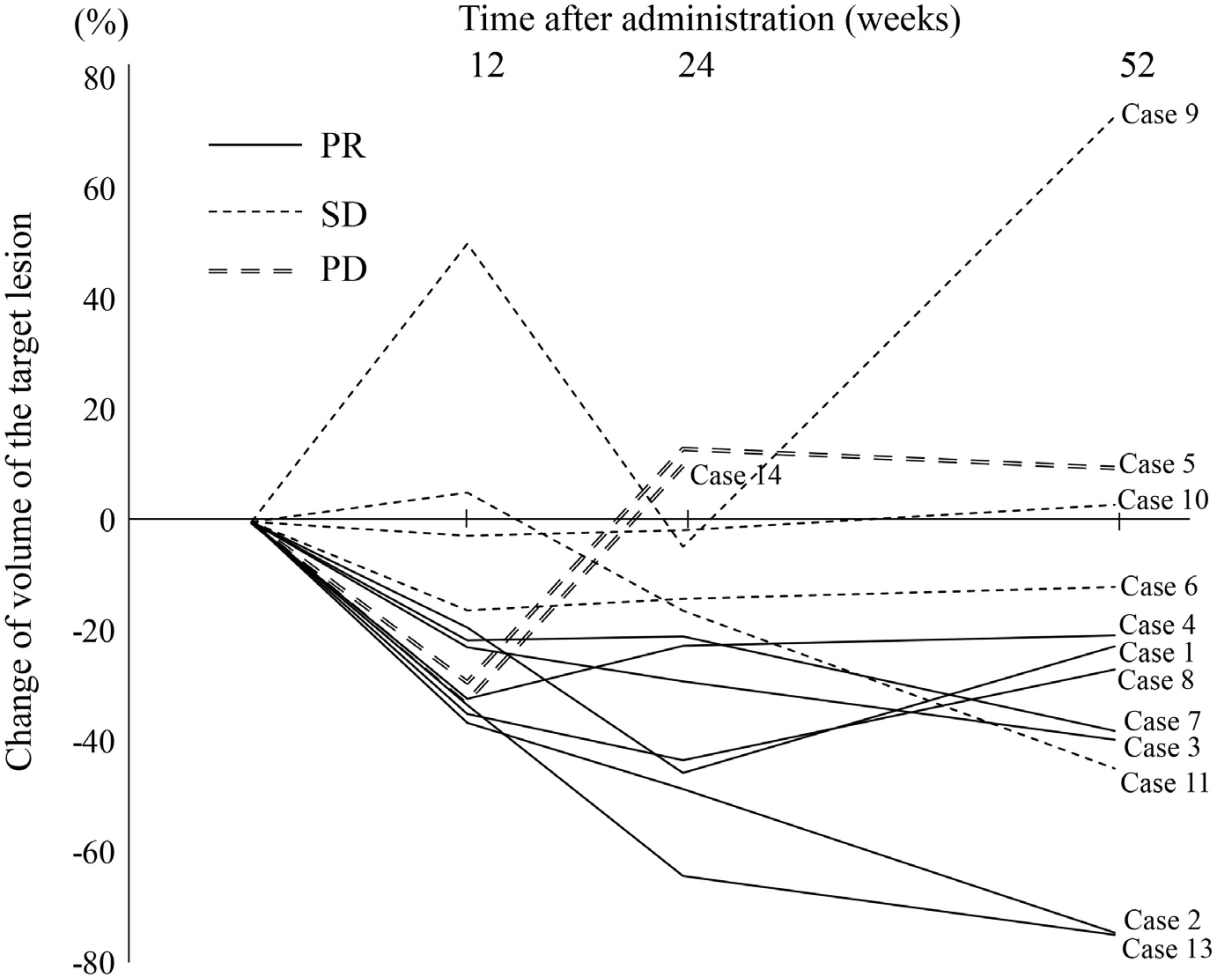
Changes in volume of the target lesions during sirolimus treatment. PR, partial response; SD, stable disease.

The detailed results for the individual cases are summarized in the Supporting Information (Table S1‐13 and Figure S1‐13). The radiological response rate at 24 weeks was 53.8% (7/13; 95% CI: 25.1%–80.8%). No patients achieved CR. Eight patients (61.5%) showed a PR at 12 weeks, and the volumes of half of the lesions were further reduced at 24 weeks. Two patients (Cases 5 and 14) also showed reductions (−29.0% and − 32.4%) at 12 weeks, but the volumes increased again at 24 weeks and they showed PD (Figure 2). In the patient with axillary cystic LM (Case 5), lymphorrhea decreased following sirolimus treatment, but lymph fluid had accumulated inside the cyst. Another patient with cervical cystic LM (Case 14) developed bleeding in the affected area before imaging evaluation. In contrast, two patients with SD (Cases 6 and 10) showed little or no reduction after 12 weeks of treatment. The radiological response rates at 24 weeks were 50.0% (2/4; 95% CI: 6.8%–93.2%) in patients administered sirolimus tablets and 55.6% (5/9; 95% CI: 21.2%–86.3%) in patients administered granules. According to disease group, the radiological response rates were 100.0% (1/1; 95% CI: 2.5%–100.0%) in patients with vascular tumors (1 KHE), 25.0% (1/4; 95% CI: 0.6%–80.6%) in patients with lymphatic disorders (3 cystic LM and 1 GLA), 66.7% (2/3; 95% CI: 9.4%–99.2%) in patients with VM (2 BRBNS and 1 VM), and 60.0% (3/5; 95% CI: 14.7%–94.7%) in patients with mixed vascular malformations (3 KTS and 2 mixed vascular malformations).

Changes in skin lesions, bleeding, blood coagulation tests, pain scale, PS, and QOL scores during sirolimus treatment are summarized in Table 3. At 12 weeks after the start of administration, 0, 2, and 2 of 12 cases showed marked improvement, improvement, and slight improvement in skin lesions, respectively, compared with 1, 3, and 2 of 11 cases at 24 weeks, and 1, 4, and 1 of 12 evaluated cases at 52 weeks. There was one case of slight worsening at 52 weeks after the start of administration. Of the five patients who experienced bleeding before treatment, three showed improvement at 24 weeks while two remained unchanged. No patients without bleeding before treatment developed new bleeding. Bleeding in the skin, soft tissues, muscles, and bones was observed in 3 of the 13 patients before treatment (grade 1 in two cases and grade 2 in one case according to the WHO‐Bleeding Scale). Two patients showed improvement after treatment, while one remained unchanged.

**TABLE 3:** Changes in skin lesions, bleeding, blood coagulation tests, pain scale, PS, and QOL scores during sirolimus treatment.

	Pre‐treatment	12 weeks	24 weeks	52 weeks
Skin lesions^a^				
Improvement (marked improvement, improvement, and slight improvement)		4/12 (33.3%), [9.9–65.1]^c^	6/11^b^ (54.5%), [23.4–83.3]^c^	6/12 (50.0%), [21.1–78.9]^c^
Bleeding				
None	8/13 (61.5%)	11/13 (84.6%)	10/12 (83.3%)	12/13 (92.3%)
Grade 1	3/13 (23.1%)	1/13 (7.7%)	1/12 (8.3%)	0/13 (0%)
Grade 2	2/13 (15.4%)	1/13 (7.7%)	1/12 (8.3%)	1/13 (7.7%)
Blood coagulation tests^d^				
Platelets (×10^4^/μL)	8.8 (5.5–12.0)^e^ (*n* = 2)	21.4 (16.2–26.5)^e^ (*n* = 2)	29.6 (19.2–40.0)^e^ (*n* = 2)	28.0 (16.4–39.6)^e^ (*n* = 2)
Fibrinogen (mg/dL)	167 (79–192)^e^ (*n* = 7)	255 (91–362)^e^ (*n* = 7)	219 (78–354)^e^ (*n* = 6)	217 (58–287)^e^ (*n* = 7)
D‐dimer (μg/mL)	13.3 (1.4–43.9)^e^ (*n* = 8)	2.2 (0.8–44.0)^e^ (*n* = 8)	2.3 (0.5–36.5)^e^ (*n* = 8)	1.7 (0.5–33.1)^e^ (*n* = 8)
Pain scale^f^				
	1.0 (0–68)^e^ (*n* = 8)	4.5 (0–72)^e^ (*n* = 8)	26.0 (0–83)^e^ (*n* = 7)	1.0 (0–92)^e^ (*n* = 8)
QOL scores				
PedsQL (total scores) (*n* = 12)	73.0 (18.5–100)^e^	89.1 (50–100)^e^	86.7 (41.7–100)^e^	95.0 (52.2–100)^e^
FACT‐G (total scores) (*n* = 1)	81.3	83.3	78.0	76.0
PS scores				
Karnofsky (*n* = 4)				
60	1/4 (25.0%)	1/4 (25.0%)	1/3 (33.3%)	1/4 (25.0%)
70	0	0	0	0
80	3/4 (75.0%)	1/4 (25.0%)	1/3 (33.3%)	1/4 (25.0%)
90	0	2/4 (50.0%)	1/3 (33.3%)	2/4 (50.0%)
100	0	0	0	0
Lansky (*n* = 9)				
60	1/9 (11.1%)	0	0	0
70	4/9 (44.4%)	0	0	0
80	0	2/9 (22.2%)	0	0
90	2/9 (22.2%)	5/9 (55.6%)	7/9 (77.8%)	7/9 (77.8%)
100	2/9 (22.2%)	2/9 (22.2%)	2/9 (22.2%)	2/9 (22.2%)
Combined				
60	2/13 (15.4%)	1/13 (7.7%)	1/12 (8.3%)	1/13 (7.7%)
70	4/13 (30.8%)	0	0	1/13 (7.7%)
80	3/13 (23.1%)	3/13 (23.1%)	1/12 (8.3%)	0
90	2/13 (15.4%)	7/13 (53.8%)	8/12 (66.7%)	9/13 (69.2%)
100	2/13 (15.4%)	2/13 (15.4%)	2/12 (16.7%)	2/13 (15.4%)

Abbreviations: CI, confidence interval; VAS, visual analog scale; QOL, quality of life; FACT‐G, Functional Assessment of Cancer Therapy‐General; PS, performance status.

^a^ Data for patients with evaluable skin lesions before treatment.

^b^ Case 1 could not be evaluated at the 24‐week time point.

^c^ 95% CI.

^d^ Data for patients with abnormal coagulation parameters before treatment.

^e^ Median (minimum–maximum).

^f^ Data for patients with evaluable VAS before treatment.

Among the subjects with abnormal blood coagulation parameters at baseline, platelet counts were normalized in 50.0% (1/2), 100.0% (2/2), and 50.0% (1/2) at 12, 24, and 52 weeks, respectively, fibrinogen was normalized in 57.1% (4/7), 66.7% (4/6), and 71.4% (5/7), respectively, and D‐dimer in 12.5% (1/8), 50.0% (4/8), and 37.5% (3/8), respectively. Some parameters were not normalized, but some improvements were noted. Although no significant differences were observed before and after treatment, the mean fibrinogen level increased after treatment and exceeded the baseline value at all observation points, while the mean D‐dimer level decreased below the baseline value at all observation points after treatment commencement (Table 3).

There were no significant differences in pain scales before and after treatment. Regarding QOL scores, there were no significant changes in the overall PedsQL (12 patients aged <25 years) or FACT‐G scores (1 patient aged ≥26 years) between the pre‐treatment period and 24 weeks of treatment. The adjusted mean changes (95% CI) from baseline in total PedsQL scores at 12, 24, and 52 weeks after the start of administration (*n* = 12) were 8.40 (−1.63 to 18.44), 10.47 (−2.82 to 23.76), and 12.75 (2.64 to 22.86), respectively. Likewise, there was no significant improvement in activities of daily living based on the pre‐treatment and post‐treatment Karnofsky PS (4 cases) and Lansky play‐PS scores (8 cases). The combined PS scores were increased compared with baseline in seven patients (7/13, 53.8%) at 12 weeks, six patients (6/12, 50.0%) at 24 weeks, and seven patients (7/13, 53.8%) at 52 weeks. The change in score from baseline at 24 weeks was 0 in six patients (6/12, 50.0%), 10 in one patient (1/12, 8.3%), 20 in four patients (4/12, 33.3%), and 30 in one patient (1/12, 8.3%), indicating a significant change (*p* = 0.031).

### Sirolimus treatment and drug concentrations

**FIGURE 3:**
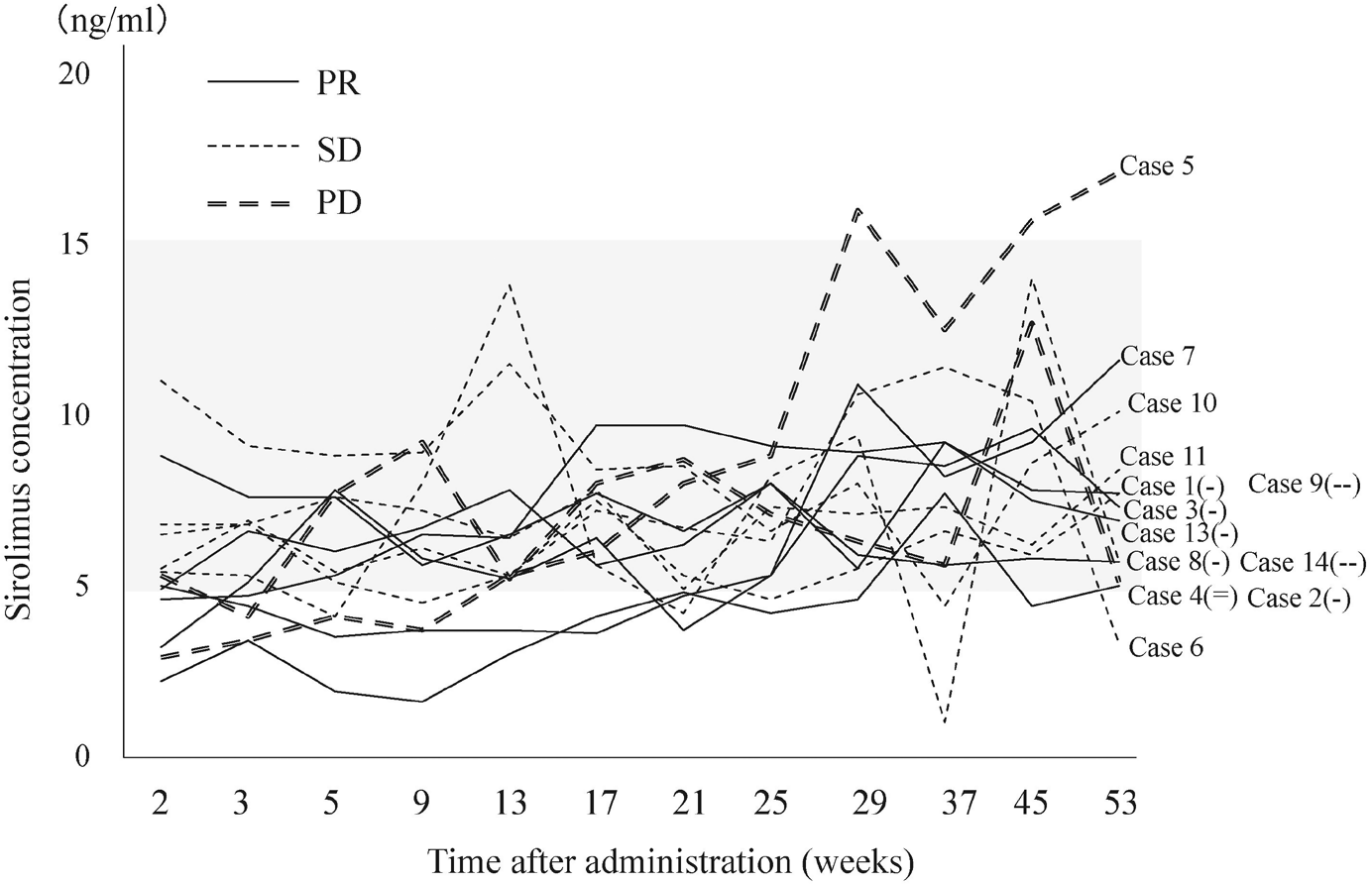
Changes in sirolimus concentrations during treatment. Gray band indicates target trough sirolimus concentration range (5–15 ng/mL). PR, partial response; SD, stable disease.

The sirolimus concentration was within the target range at nearly all measurement points (Figure 3): 6.6 ± 3.0 ng/mL at 12 weeks after the start of administration, 6.6 ± 1.5 ng/mL at 24 weeks, and 7.8 ± 3.6 ng/mL at 52 weeks. There was no notable difference in sirolimus concentrations between patients with PR and patients with SD. The trough concentration did not exceed 5 ng/mL in two cases (Cases 2 and 3) at 21 weeks, both of whom had taken the granule formulation; however, these low concentrations were not correlated with the effectiveness of sirolimus, and the concentrations in these patients varied throughout the rest of the treatment period.

### Safety

Adverse events were observed in all 13 subjects, but none exceeded grade 4. Grade 3 adverse events were observed in nine subjects (69.2%). There were no fatal adverse events. Stomatitis was the most common side effect, occurring in 10 patients (76.9%), followed by fever in nine (69.2%), diarrhea in four (30.8%), and acneiform dermatitis and neutropenia in three each (23.1%). Other events included abdominal pain, nausea, fatigue, upper respiratory tract infection, respiratory syncytial viral infections, rhinorrhea, upper respiratory tract inflammation, and headache in two patients (15.4%). All other adverse events were singular occurrences. There was no difference in the frequency of side effects between the different drug forms.

## DISCUSSION

In this study, sirolimus was administered for various types of VAs. The small number of cases meant that it was challenging to evaluate each disease group separately; however, we analyzed the results by dividing the patients into two categories: vascular tumors and vascular malformations.

KHE, a rare vascular tumor associated with the Kasabach–Merritt phenomenon (KMP), lacks standardized treatment approaches. We observed rapid improvement in KMP and a clear reduction in tumor volume in the single affected patient in the current trial, consistent with previous reports. Ji et al. recently conducted a randomized clinical trial in patients with KMP comparing sirolimus monotherapy (0.8 mg/m^2^/day, target trough concentration: 10–15 ng/mL) with a combined short‐term prednisolone regimen (2 mg/kg/day).^12^ The combination therapy group showed superior outcomes, including a higher rate of platelet recovery (94.6% vs. 66.7%), a reduced transfusion rate (1.4% vs. 2.5%), and a decreased risk of recurrence (5.4% vs. 16.7%), with no significant difference in adverse effects between the groups. Although our case showed positive results with monotherapy, a combination approach might offer enhanced benefits and reduce recurrence risk. This highlights the need for more research, particularly in relation to stratified treatments based on disease severity.

Somatic *PIK3CA* mutations are common in LM, VM, KTS, and other VAs.^2^ The relationship between sirolimus efficacy and the presence of *PIK3CA* gene mutations is currently unclear; however, sirolimus could have positive therapeutic effects by inhibiting signals activated by genetic mutations in this pathway.^3^, ^4^ Sirolimus was previously shown to be highly effective in patients with VM, LM, and combined lesions, resulting in a PR in all patients, reducing symptoms, and increasing QOL.^5^, ^6^, ^13^ Patients in the current trial were not required to undergo genetic analysis. Biopsy or removal of the lesion carries risks of severe bleeding, infection, and postoperative lymph leakage, and genetic analysis before sirolimus treatment is challenging in clinical practice. Moreover, the detection sensitivity for genetic mutations from lesions is currently low, and the process is time‐consuming and costly. At the time of planning the current study, there was no evidence linking genetic mutations to the effectiveness of sirolimus treatment. Further research is therefore needed to understand the relationship between the patient's genetic background and sirolimus treatment efficacy.

A sirolimus syrup formulation (Rapamune 1 mg/mL oral solution; Pfizer Inc., NY, USA) has been globally available. In this study, we developed and used a granule formulation of sirolimus. Sirolimus tablets were approved in Japan for lymphangioleiomyomatosis in 2014 and for intractable LMs in 2021.^13^ Sirolimus was initially only available in tablet form, which is suitable for adults and older children; however, administering tablets to infants and patients with tube feeding is challenging, often requiring the medication to be crushed and thus raising concerns about drug solubility and stability. Use of a granule formulation has resolved these issues. We subsequently calculated the initial dosage for infants in our trial based on pharmacokinetic data from healthy Japanese adults and children for both the tablet and granule forms. Recent research indicates that sirolimus clearance continues to change until the age of 2 years, with clearance being proportional to age, and Mizuno et al. accordingly proposed estimated age‐appropriate starting dosages for sirolimus targeting specific ranges.^14^ The initial dose in the current study was based on the recommended dosing regimen, with careful adjustments according to observed trough levels. More studies and cases are needed to establish guidelines for the safe use of sirolimus in pediatric patients. Although there were no clear differences in efficacy and safety between the tablet and granule forms in the present study, further analyses with more cases are needed.

We recently conducted a population pharmacokinetic analysis using data from 215 healthy Japanese subjects and patients with VAs and other diseases administered oral sirolimus (tablets and granules).^15^ The results suggested that the granule formulation had a 1.23‐fold higher exposure than the tablet formulation. In the current study, if a change in formulation was requested at 25 weeks after administration, the patient could switch to the other formulation, and the dose was adjusted on the basis that 1 mg of the tablet formulation was equivalent to 0.7 mg of granules. Two patients (Cases 1 and 5) switched to another formulation, and their doses were adjusted according to the trough concentration. Considering the wide interindividual variability in sirolimus pharmacokinetic parameters, the dosage should be adjusted according to the target trough concentration and the patient's condition after switching.

This study had several limitations. First, the number of participants was low. In addition, the open‐label, single‐arm design could have introduced bias in evaluating certain outcomes, and the results should ideally be confirmed in a randomized, placebo‐controlled, double‐blind clinical trial; however, the rarity and heterogeneity of the diseases means that conducting such trials presents considerable challenges. A nationwide survey by Japanese researchers specializing in rare diseases during the 2010s indicated a very limited number of patients with intractable conditions, resulting in few potential participants in Japan. Enrolling suitable cases within the trial period was thus a challenge, and few patients could access the trial sites and meet the inclusion criteria. Conducting high‐evidence‐level trials for such rare diseases thus presents a formidable challenge. Moreover, there are currently no standardized evaluation methods for VAs, complicating the assessment of sirolimus treatment efficacy. The current sample size was calculated to assess the radiological response rate as the primary outcome; however, the sample size might not have been ideal for evaluating secondary outcomes. Despite clinical symptom improvements, it is possible that there were no significant changes in many secondary outcomes, including QOL.

In conclusion, the current clinical trial assessed the effectiveness, safety, and pharmacokinetics of sirolimus granules and tablets for the treatment of intractable VAs. There was no significant difference in effectiveness or safety between the two formulations. Further clinical trials with more patients are needed to verify these findings.

## AUTHOR CONTRIBUTIONS

M.O., R.A., H.H., T.M., and A.F. conceived the study and participated in its design. R.A. performed progress management and adjustment of the overall clinical trial. M.O., S.E., S.Y., M.K., T.K., T.F., S.F., T.T., J.T., and N.K. oversaw the operations associated with this clinical trial, including managing and instructing sub‐investigators and trial collaborators. H.H. was responsible for statistical analysis. A.S. was responsible for case enrollment, data management, and monitoring. S.U., S.W., M.K., K.D., S.N., M.M., A.U., K.M., and S.H. evaluated the efficacy and safety of this study. All authors read and approved the final manuscript.

## FUNDING INFORMATION

M.O. received research funding from Nobelpharma Co. Ltd. in Tokyo. Sirolimus tablets and granules were supplied by Nobelpharma Co. Ltd. The other authors declare no competing interests. The present study was supported in part by a Practical Research Project for Rare/Intractable Diseases (JP22ek0109515) from the Japan Agency for Medical Research and Development, AMED. This study has been registered with the Clinical Trial Registry (UMIN‐CTR) (A multicenter, phase 3 study assessing efficacy and safety of the Sirolimus (Granules and Tablets) in the Treatment of intractable vascular anomalies, UMIN000038973, https://center6.umin.ac.jp/cgi‐open‐bin/ctr_e/ctr_view.cgi?recptno=R000044448).

## Supporting information


Figure S1.

Figure S2.

Figure S3.

Figure S4.

Figure S5.

Figure S6.

Figure S7.

Figure S8.

Figure S9.

Figure S10.

Figure S11.

Figure S12.

Figure S13.

Table S1.
