# Supplementary figures and images for: Sirolimus treatment for intractable vascular anomalies (SIVA): An open‐label, single‐arm, multicenter, prospective trial
Source: Pediatr Int. 2025 Mar 26;67(1):e70002. doi: 10.1111/ped.70002 (PMC11937875; doi:10.1111/ped.70002)

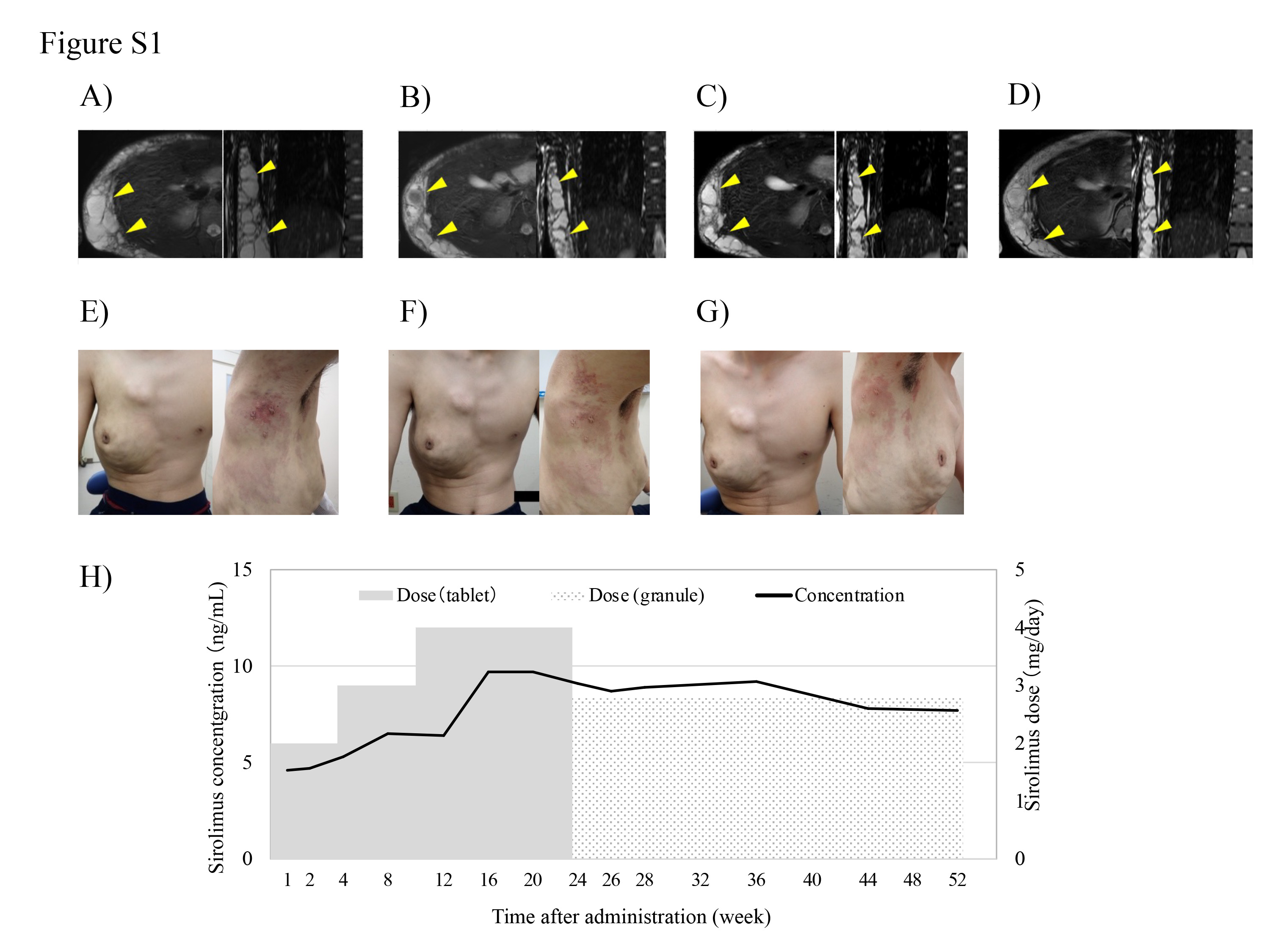

Supplement: Supplementary file 1 — Figure S1. [file PED-67-e70002-s005.jpg]

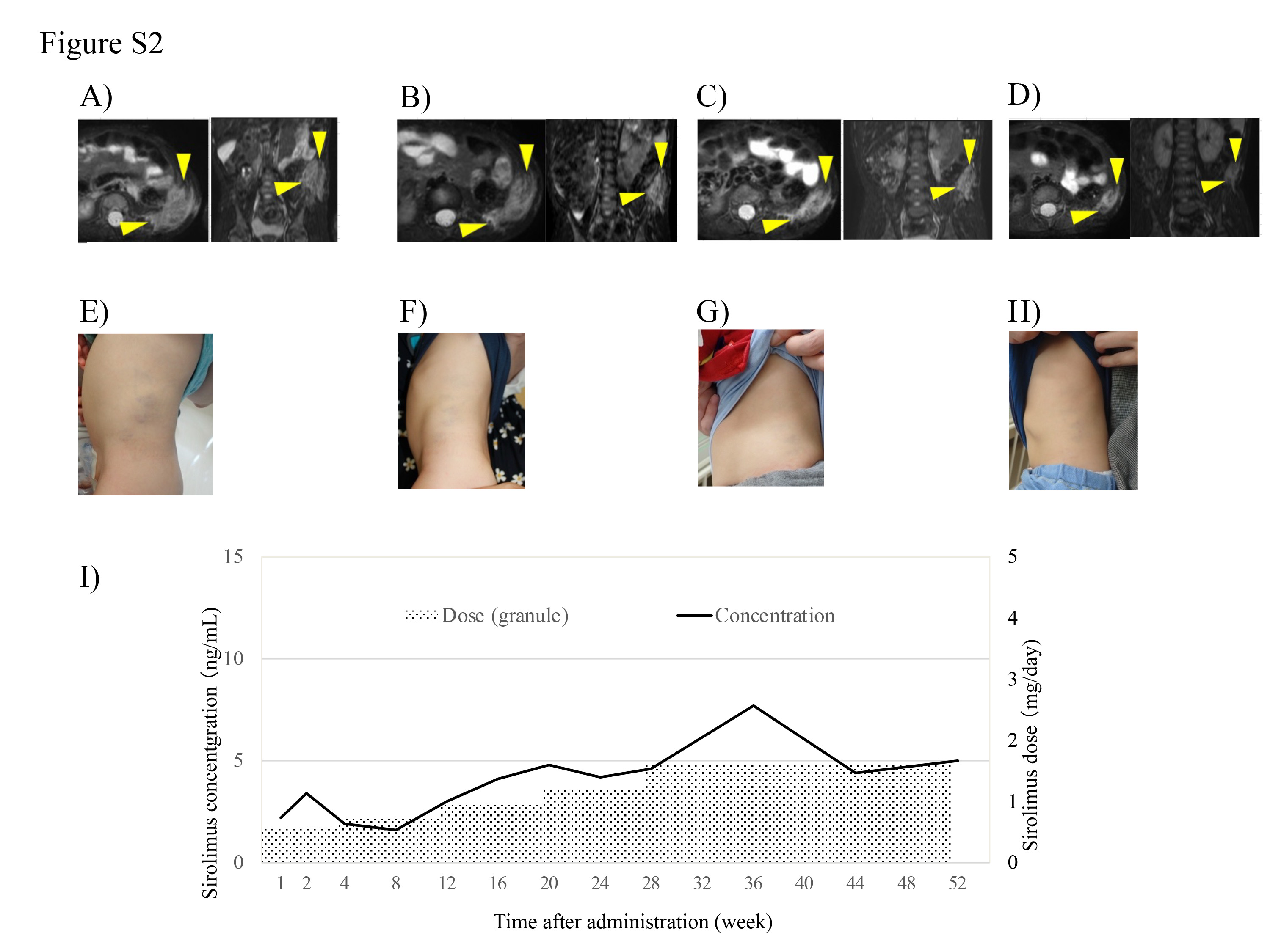

Supplement: Supplementary file 2 — Figure S2. [file PED-67-e70002-s003.jpg]

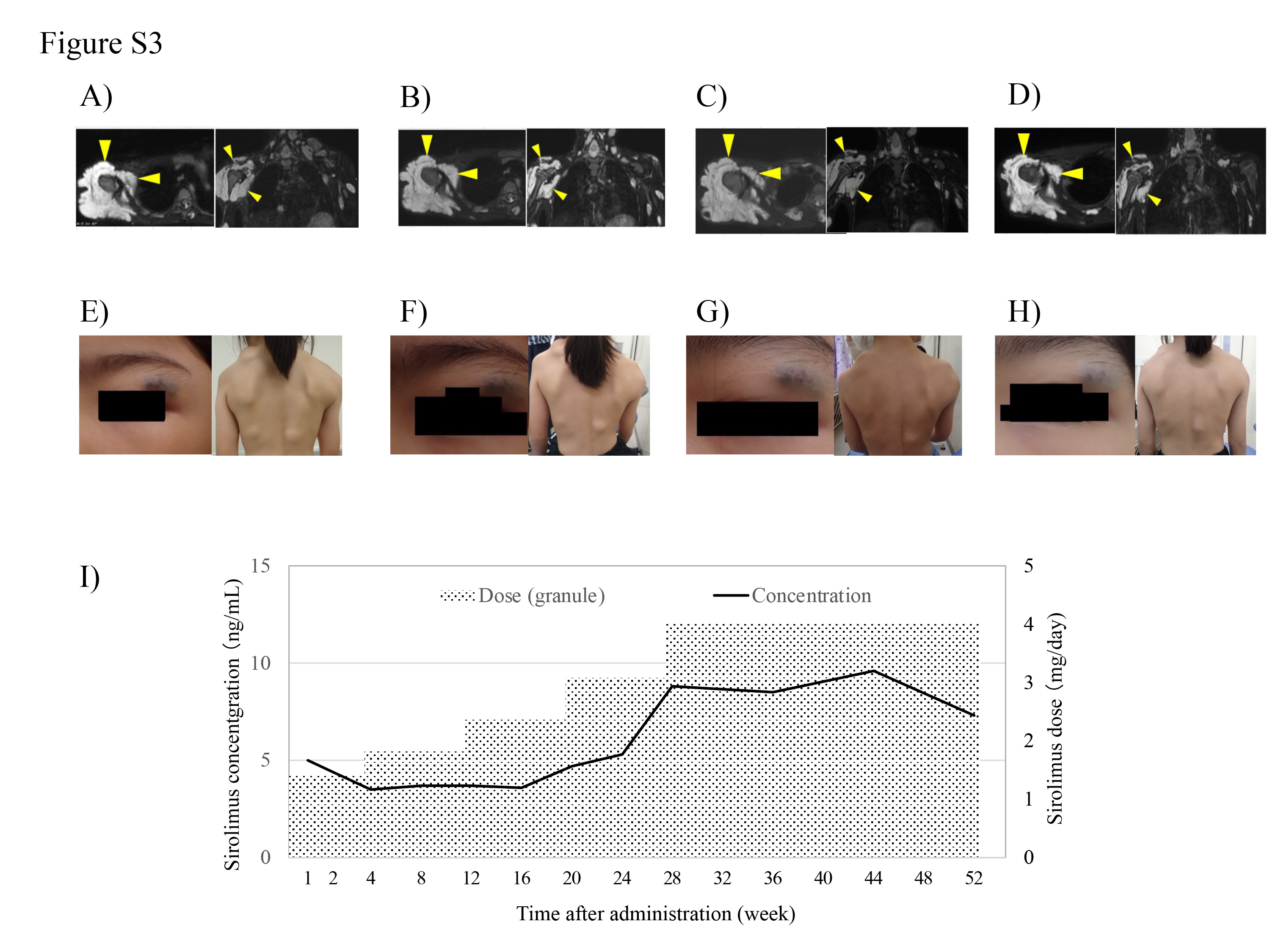

Supplement: Supplementary file 3 — Figure S3. [file PED-67-e70002-s012.jpg]

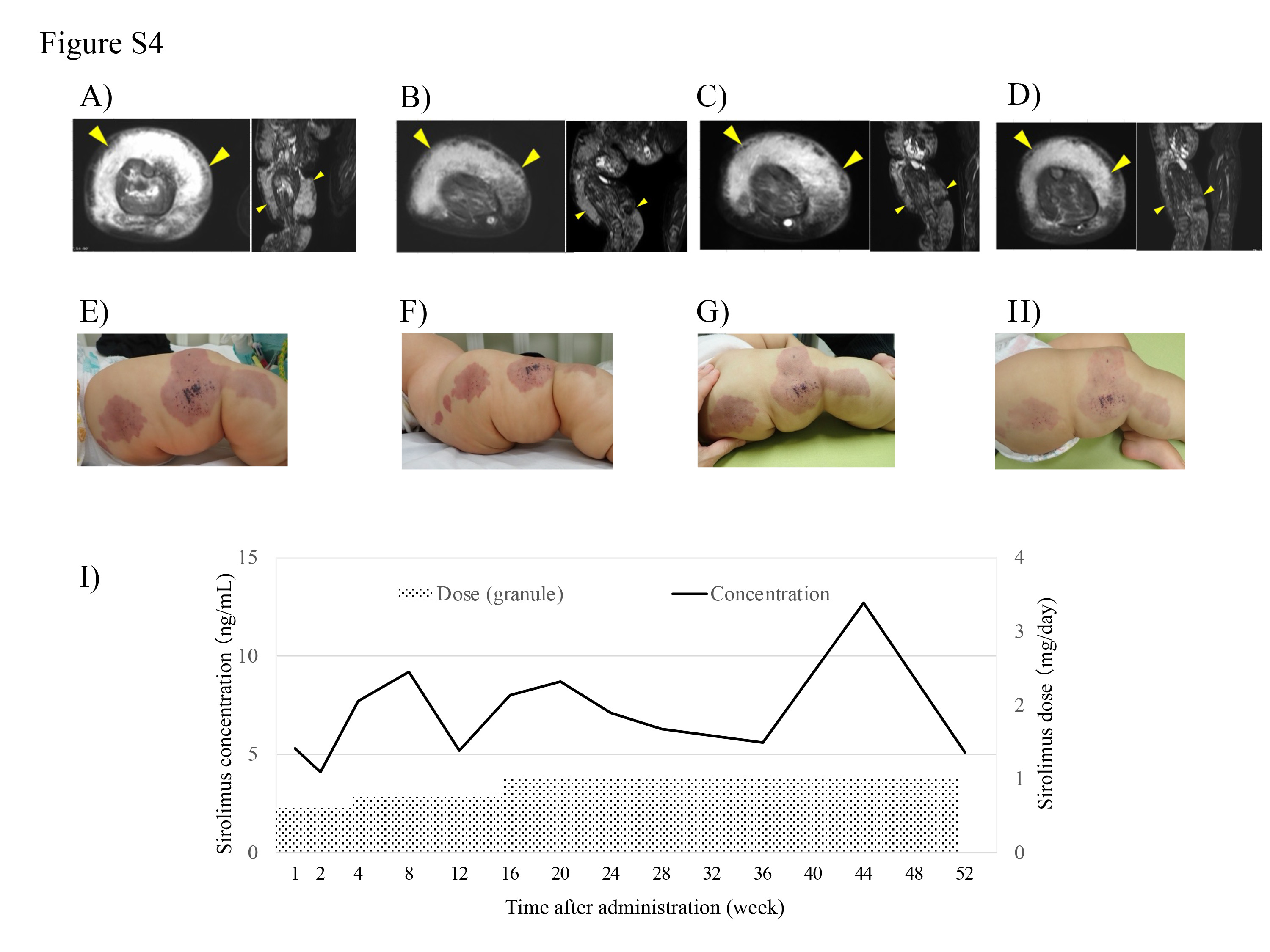

Supplement: Supplementary file 4 — Figure S4. [file PED-67-e70002-s008.jpg]

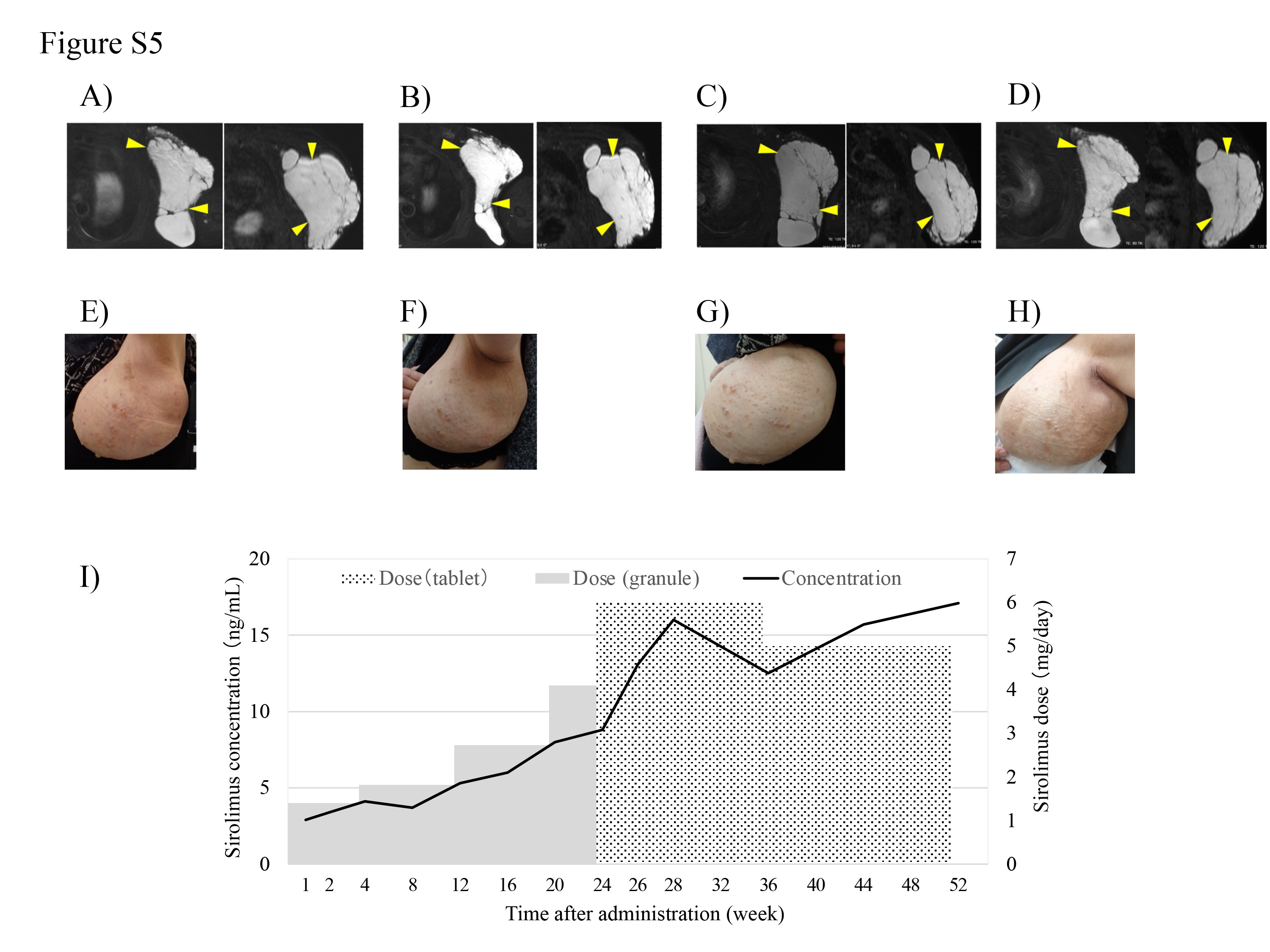

Supplement: Supplementary file 5 — Figure S5. [file PED-67-e70002-s011.jpg]

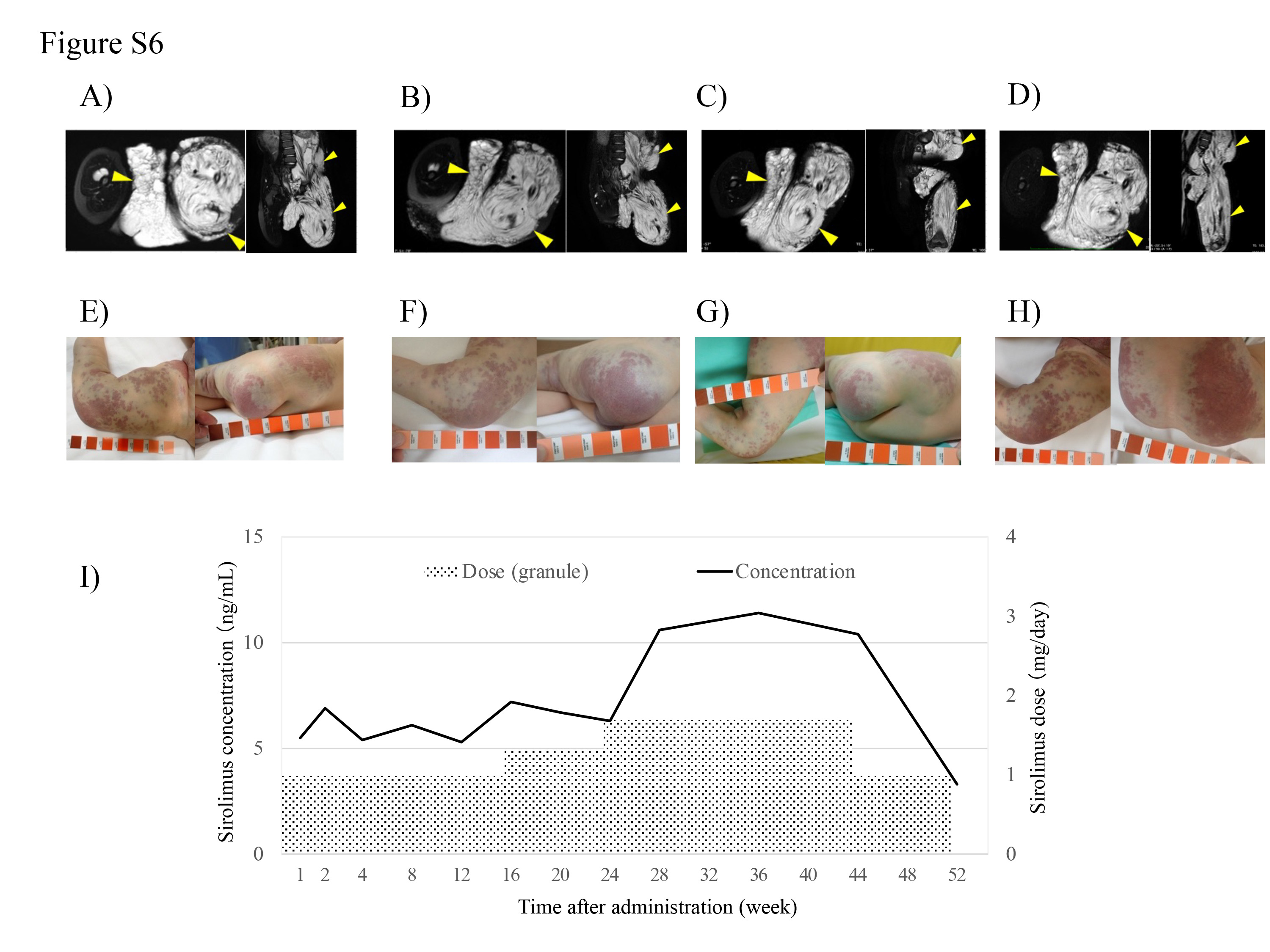

Supplement: Supplementary file 6 — Figure S6. [file PED-67-e70002-s001.jpg]

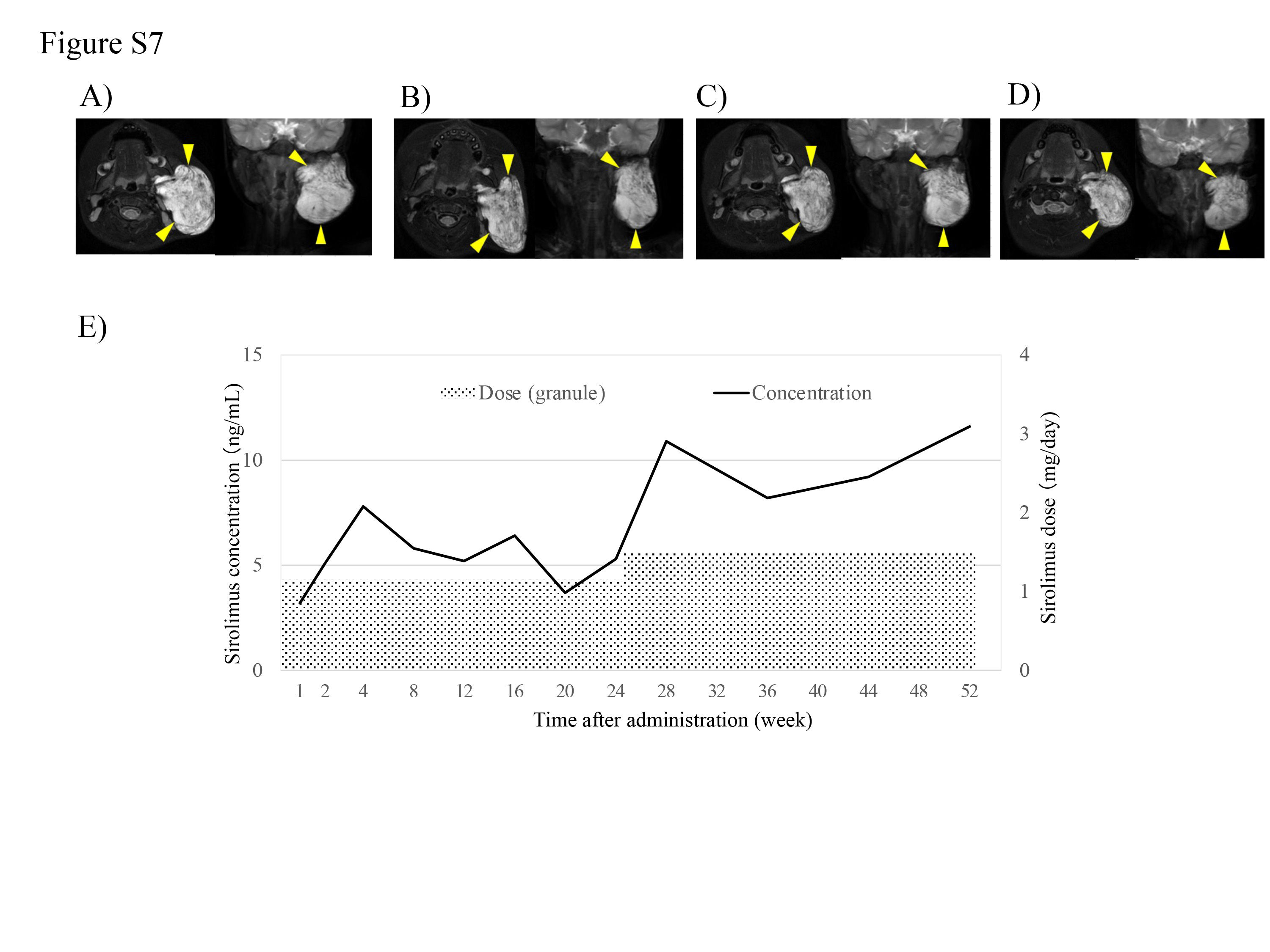

Supplement: Supplementary file 7 — Figure S7. [file PED-67-e70002-s007.jpg]

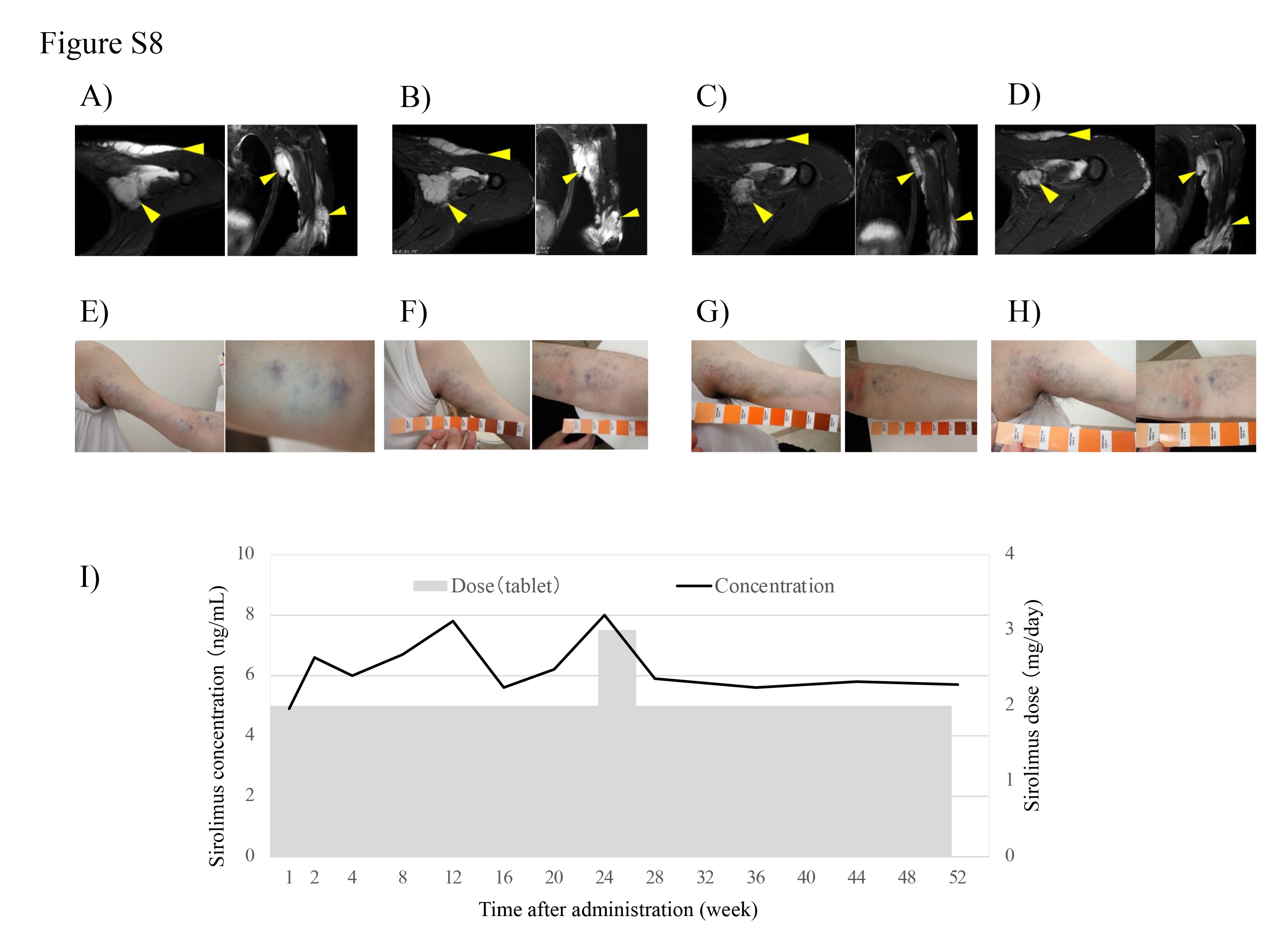

Supplement: Supplementary file 8 — Figure S8. [file PED-67-e70002-s014.jpg]

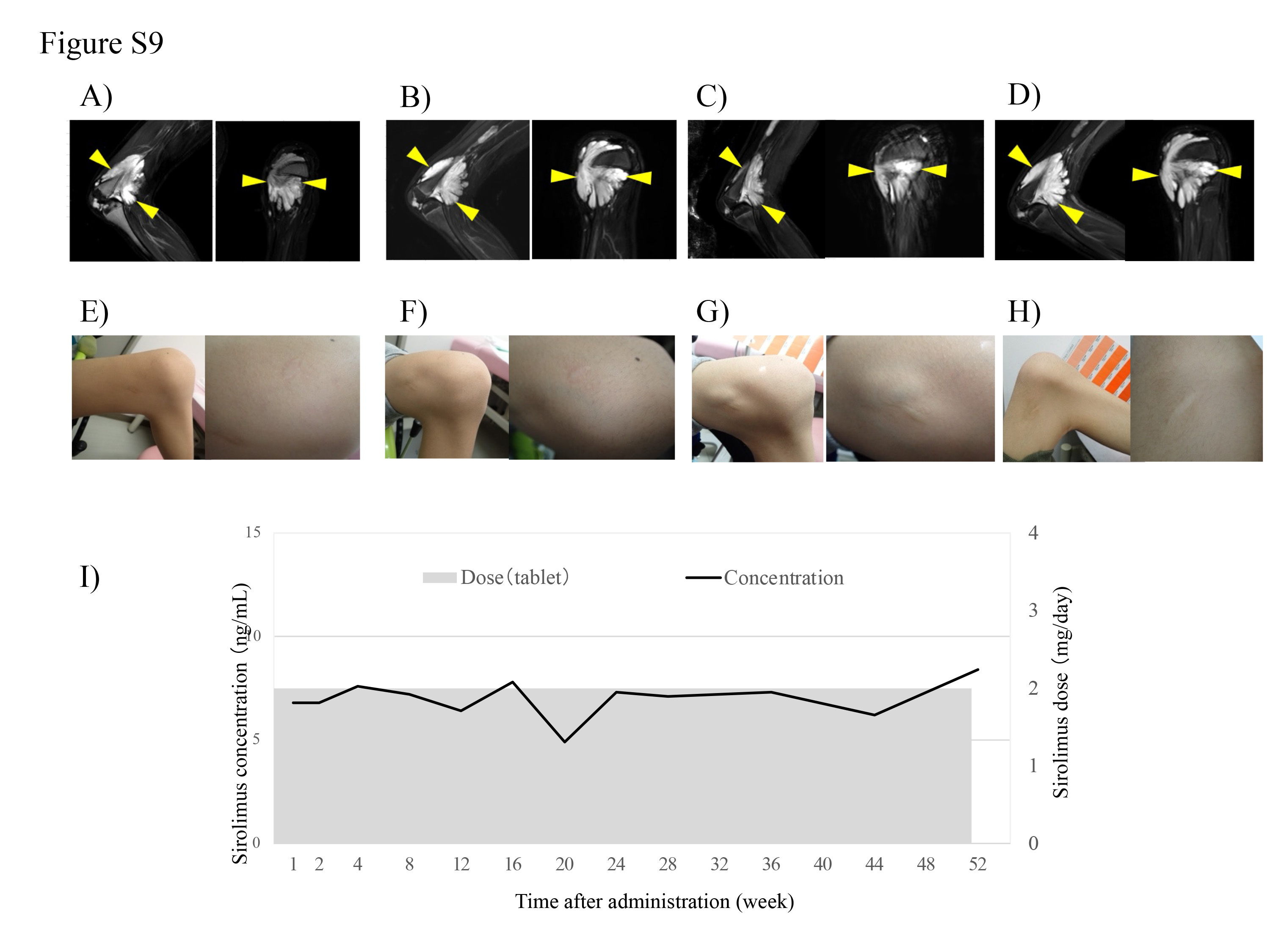

Supplement: Supplementary file 9 — Figure S9. [file PED-67-e70002-s004.jpg]

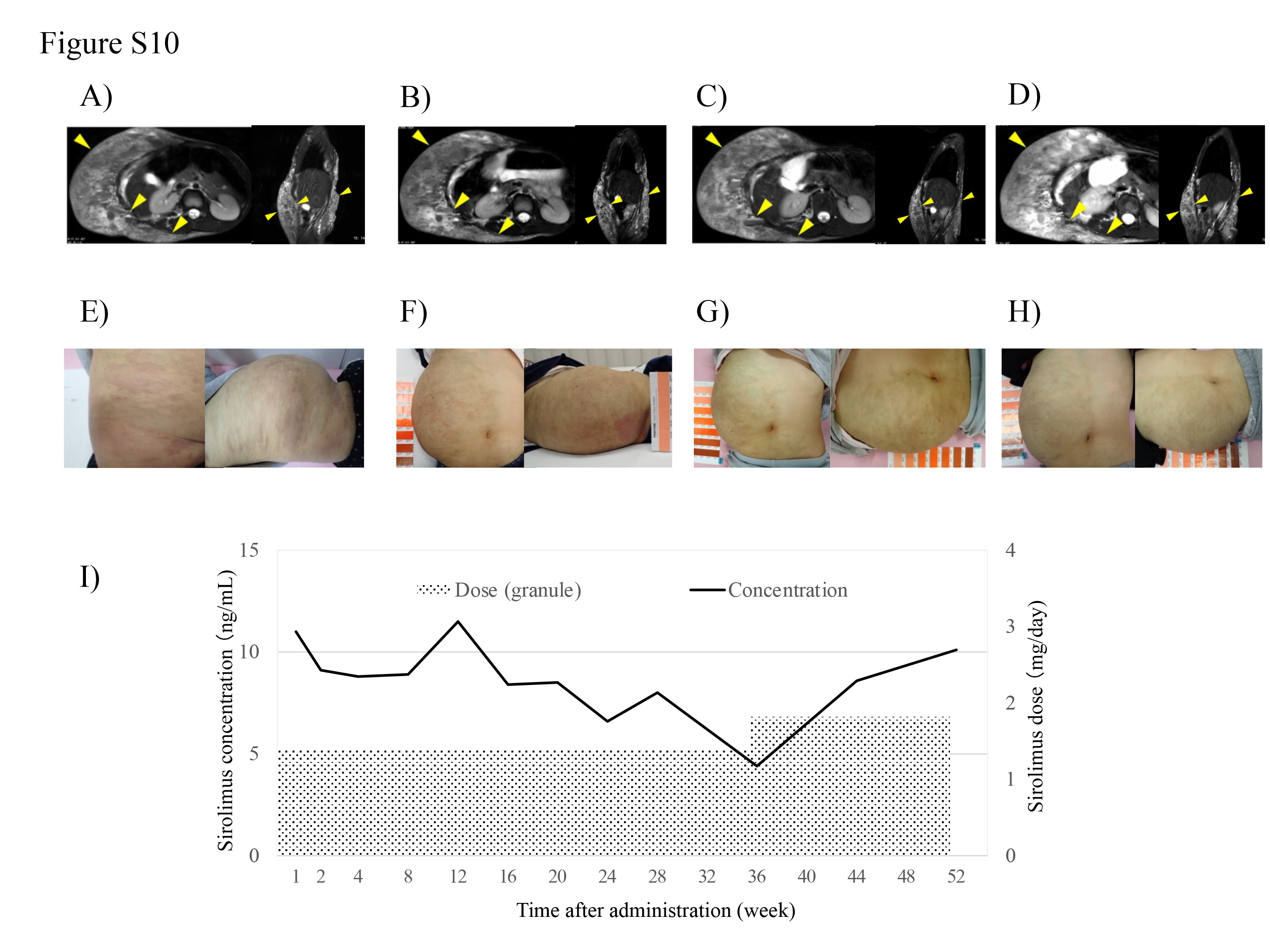

Supplement: Supplementary file 10 — Figure S10. [file PED-67-e70002-s002.jpg]

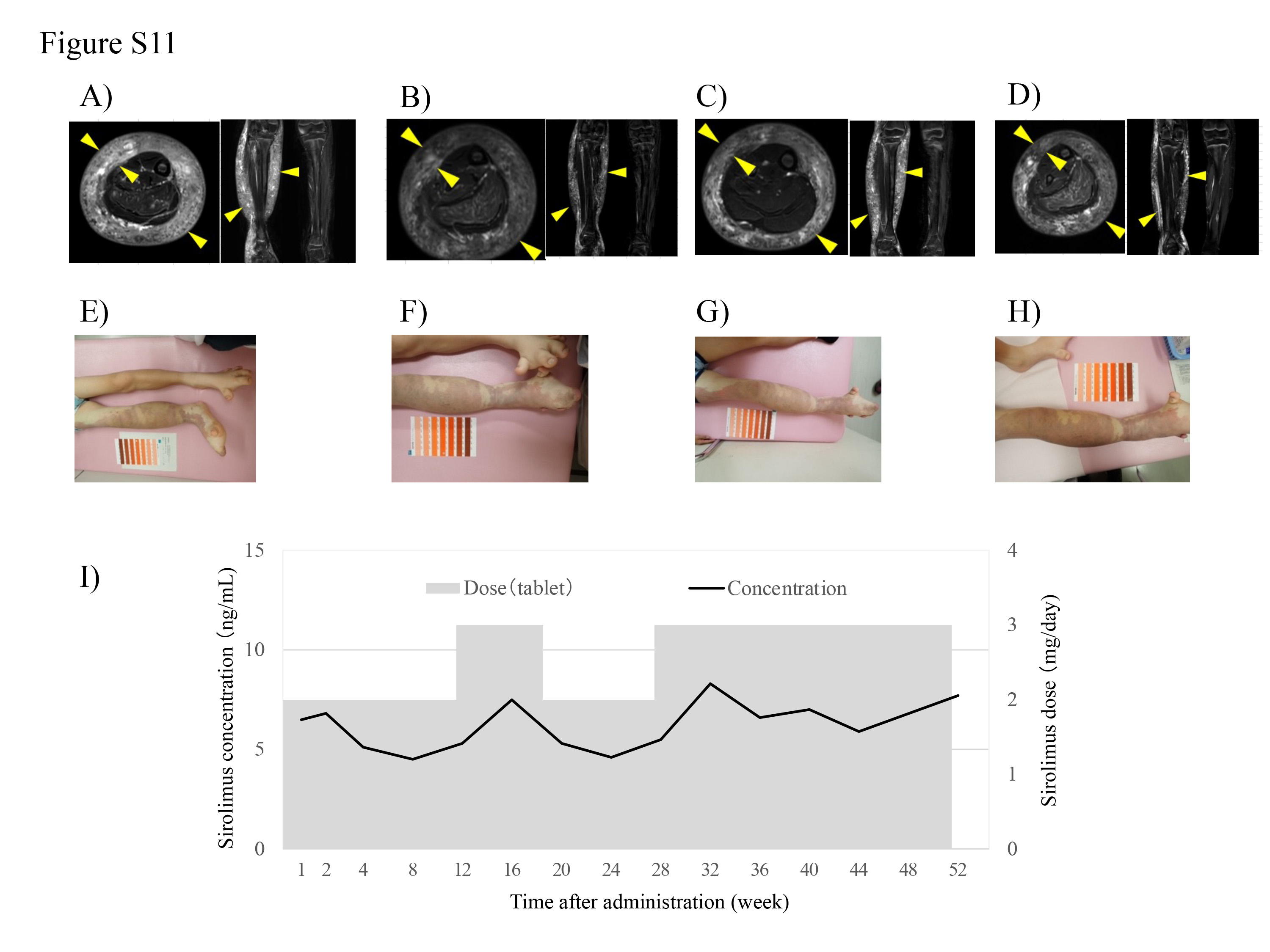

Supplement: Supplementary file 11 — Figure S11. [file PED-67-e70002-s013.jpg]

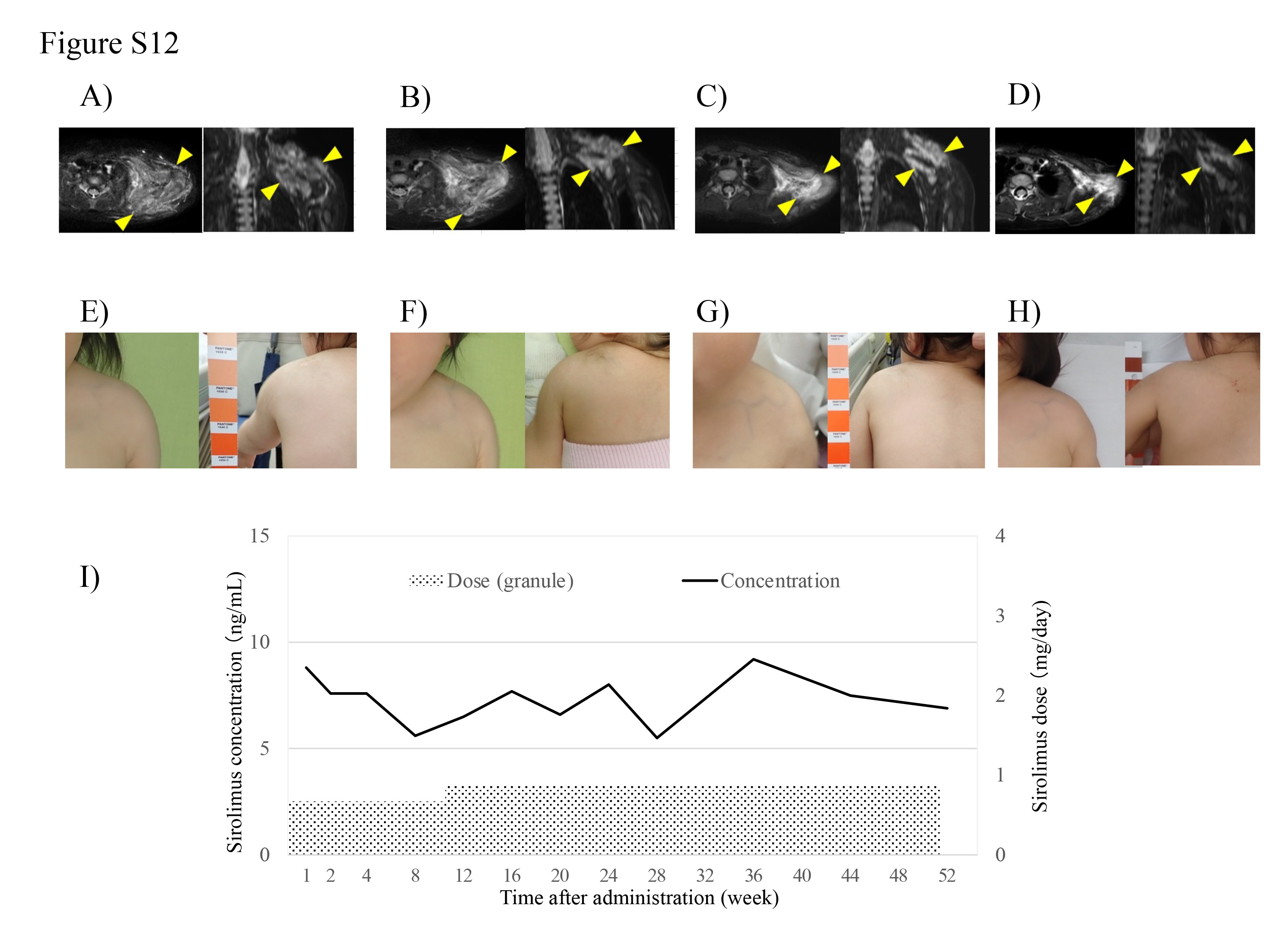

Supplement: Supplementary file 12 — Figure S12. [file PED-67-e70002-s010.jpg]

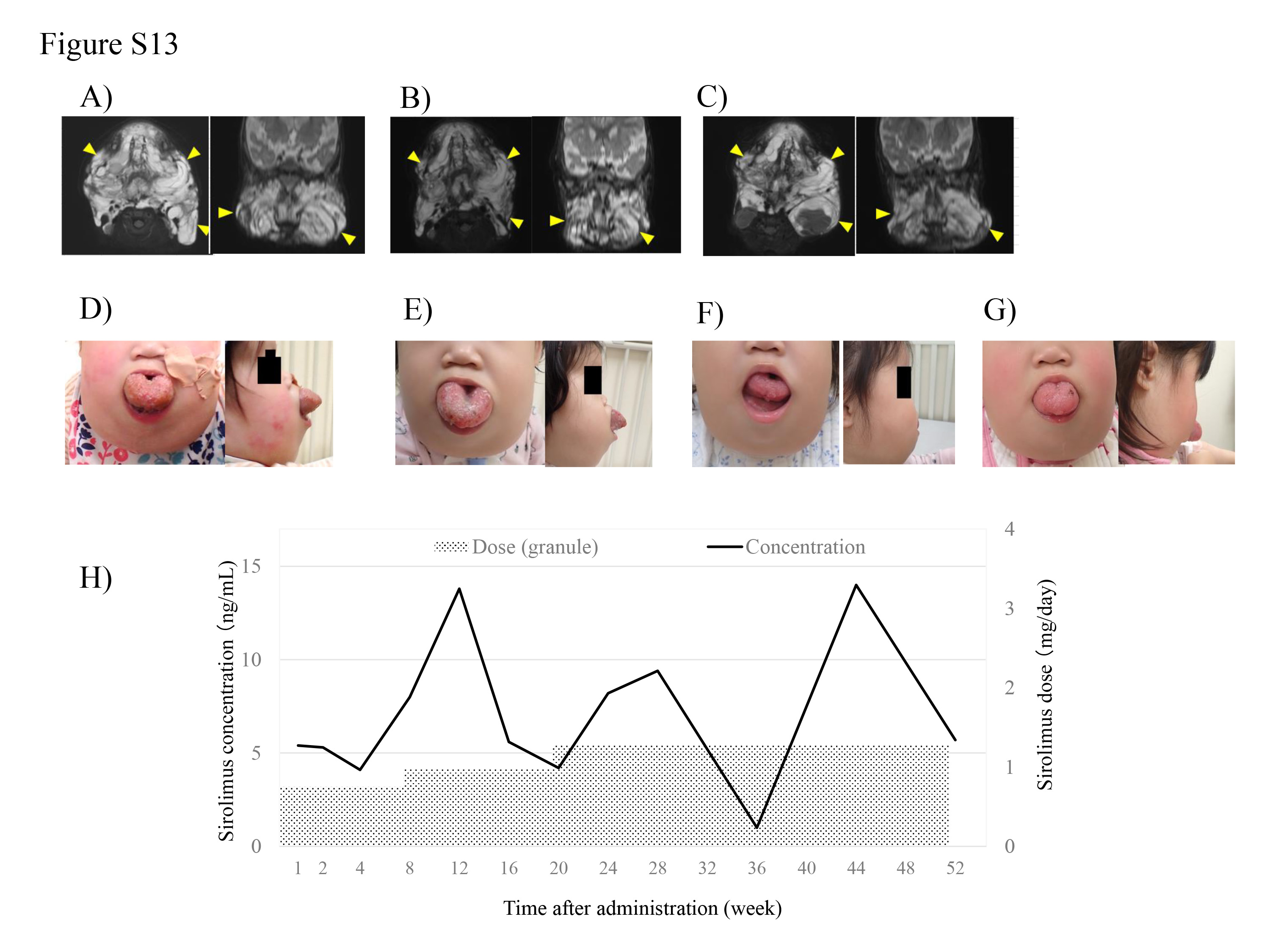

Supplement: Supplementary file 13 — Figure S13. [file PED-67-e70002-s006.jpg]
