# Supplementary material for: Sirolimus treatment for intractable vascular anomalies (SIVA): An open‐label, single‐arm, multicenter, prospective trial
Source: Pediatr Int. 2025 Mar 26;67(1):e70002. doi: 10.1111/ped.70002 (PMC11937875; doi:10.1111/ped.70002)
Supplement: Supplementary file 14 — Table S1. [file PED-67-e70002-s009.docx]

Table S1. Clinical data of case 1

a) age (years old), sex, diagnosis (sites of target lesion), height (cm), body weight (kg) and body surface area (m^2^)

b) change of volume of the target lesion and evaluation of lesions

c) secondary assessments; pleural effusion, ascites, blood coagulation test (platelet counts, fibrinogen and D-dimer), bleeding scales (the World Health Organization-Bleeding Scale), pain scales (visual analog scales), Functional Assessment of Cancer Therapy-General (FACT-G) (>25 years old)) and performance status (PS) (Karnofsky Performance Status (>10 years old), Lansky play-performance scale (for patients <10 years old), Quality of Life (QOL) scores (PedsQL™ 4.0 Generic Core Scales (< 25 years old), and change of other clinical symptoms,.

d) adverse events; events and grade (the Common Terminology Criteria for Adverse Events V4.0) divided into periods (after 0-12 weeks, 13-24 weeks, 25-52 weeks) (underline: causal relationship cannot be ruled out)

| a) 18 years old, male, complex vascular anomaly (right chest, subcutaneous), 181cm, 69.7kg, 1.87m^2^ | | | | |
| --- | --- | --- | --- | --- |
| b) | | | | |
|  | Pre-treatment | After 12 weeks | After 24 weeks | After 52 weeks |
| Change of volume of the target lesion |  | -19.1 % | -45.4 % | -22.5 % |
| Evaluation of lesions |  | Stable disease | Partial response | Partial response |
| c) | | | | |
|  | Pre-treatment | After 12 weeks | After 24 weeks | After 52 weeks |
| Pleural effusion | - | - | N.A. | - |
| Ascites | - | - | N.A. | - |
| Platelet counts (/ul) | 19.1 | 21.5 | N.A. | 25 |
| Fibrinogen (mg/dl) | 176 | 255 | N.A. | 217 |
| D-dimer (ug/ml) | 0.5 | 0.5 | N.A. | 0.8 |
| Bleeding scale | None | None | N.A. | None |
| Pain scale | 53 | 4 | N.A. | 0 |
| PS (Karnofsky) | 80 | 90 | N.A. | 90 |
| QOL (PedsQL) | 63.0 | 100.0 | 100.0 | 100.0 |
| Change of other clinical symptoms |  | Reduction of pain | - | The lesion became softer. |
| d) | | | | |
|  | 0-12 weeks | 13-24 weeks | 25-52 weeks |  |
| Events (CTCAE grade) | Stomatitis (2) | Stomatitis (2) | Acne (1) |  |

PS; performance status, QOL; Quality of Life, N.A.; not analyzed. -; no data, CTCAE; Common Terminology Criteria for Adverse Events.

Table S2. Clinical data of case 2

a) age (years old), sex, diagnosis (sites of target lesion), height (cm), body weight (kg) and body surface area (m^2^)

b) change of volume of the target lesion and evaluation of lesions

c) secondary assessments; pleural effusion, ascites, blood coagulation test (platelet counts, fibrinogen and D-dimer), bleeding scales (the World Health Organization-Bleeding Scale), pain scales (visual analog scales), Functional Assessment of Cancer Therapy-General (FACT-G) (>25 years old)) and performance status (PS) (Karnofsky Performance Status (>10 years old), Lansky play-performance scale (for patients <10 years old), Quality of Life (QOL) scores (PedsQL™ 4.0 Generic Core Scales (< 25 years old), and change of other clinical symptoms,.

d) adverse events; events and grade (the Common Terminology Criteria for Adverse Events V4.0) divided into periods (after 0-12 weeks, 13-24 weeks, 25-52 weeks) (underline: causal relationship cannot be ruled out)

| a) 1 years old, male, combined vascular anomaly (left lateral abdominal, subcutaneous), 69cm, 6.9kg, 0.36m^2^ | | | | |  |
| --- | --- | --- | --- | --- | --- |
| b) | | | | |  |
|  | Pre-treatment | After 12 weeks | After 24 weeks | After 52 weeks |  |
| Change of volume of the target lesion |  | -36.3% | -48.3% | -74.3% |  |
| Evaluation of lesions |  | Partial response | Partial response | Partial response |  |
| c) | | | | |  |
|  | Pre-treatment | After 12 weeks | After 24 weeks | After 52 weeks |  |
| Pleural effusion | - | - | - | - | |
| Ascites | - | - | - | - | |
| Platelet counts (/ul) | 5.5 | 26.5 | 40 | 39.6 | |
| Fibrinogen (mg/dl) | 158 | 362 | 303 | 267 | |
| D-dimer (ug/ml) | 22.1 | 0.8 | 0.5 | <0.5 | |
| Bleeding scale | Grade1 | None | None | None | |
| Pain scale | - | - | - | - | |
| PS (Lansky) | 70 | 90 | 90 | 90 | |
| QOL (Peds-QL) | 100.0 | 95.8 | 89.6 | 100.0 | |
| Change of other clinical symptoms |  | The lesion became softer. | Unchanged | Unchanged | |
| d) | | | | | |
|  | 0-12 weeks | 13-24 weeks | 25-52 weeks |  | |
| Events (CTCAE grade) |  | Bacterial infection (3) | allergic rhinitis (2), dry skin (2), fever (1) and allergic rhinitis (2) |  | |

PS; performance status, QOL; Quality of Life, N.A.; not analyzed. -; no data, CTCAE; Common Terminology Criteria for Adverse Events.

Table S3. Clinical data of case 3

a) age (years old), sex, diagnosis (sites of target lesion), height (cm), body weight (kg) and body surface area (m^2^)

b) change of volume of the target lesion and evaluation of lesions

c) secondary assessments; pleural effusion, ascites, blood coagulation test (platelet counts, fibrinogen and D-dimer), bleeding scales (the World Health Organization-Bleeding Scale), pain scales (visual analog scales), Functional Assessment of Cancer Therapy-General (FACT-G) (>25 years old)) and performance status (PS) (Karnofsky Performance Status (>10 years old), Lansky play-performance scale (for patients <10 years old), Quality of Life (QOL) scores (PedsQL™ 4.0 Generic Core Scales (< 25 years old), and change of other clinical symptoms,.

d) adverse events; events and grade (the Common Terminology Criteria for Adverse Events V4.0) divided into periods (after 0-12 weeks, 13-24 weeks, 25-52 weeks) (underline: causal relationship cannot be ruled out)

| a) 8 years old, female, blue rubber bleb nevus syndrome (right shoulder, subcutaneous), 117cm, 21.0kg, 0.83m^2^ | | | | |  |
| --- | --- | --- | --- | --- | --- |
| b) | | | | |  |
|  | Pre-treatment | After 12 weeks | After 24 weeks | After 52 weeks |  |
| Change of volume of the target lesion |  | -22.7% | -28.8% | -39.4% |  |
| Evaluation of lesions |  | Partial response | Partial response | Partial response |  |
| c) | | | | |  |
|  | Pre-treatment | After 12 weeks | After 24 weeks | After 52 weeks |  |
| Pleural effusion | - | - | - | - | |
| Ascites | - | - | - | - | |
| Platelet counts (/ul) | 20 | 20.2 | 19.7 | 21.7 | |
| Fibrinogen (mg/dl) | 79 | 107 | 134 | 157 | |
| D-dimer (ug/ml) | 43.9 | 32 | 36.5 | 30.3 | |
| Bleeding scale | Grade2 | Grade1 | None | None | |
| Pain scale | 68 | 48 | 26 | 8 | |
| PS (Lansky) | 70 | 80 | 90 | 90 | |
| QOL (Peds-QL) | 65.2 | 93.5 | 93.5 | 98.9 | |
| Change of other clinical symptoms |  | Improvement of anemia (Hb 9.9g/dL at pre-treatment→ 12.6g/dL) | Hb 13.0g/dL | Hb 13.6g/dL |  |
| d) | | | | |  |
|  | 0-12 weeks | 13-24 weeks | 25-52 weeks |  |  |
| Events (CTCAE grade) | Gastroenteritis (2) and stomatitis (2) | Stomatitis (1), allergic rhinitis (2), rhinorrhea　(1), and oropharyngeal pain (1) | Nausea (2) and diarrhea (2) |  |  |

PS; performance status, QOL; Quality of Life, N.A.; not analyzed. -; no data, CTCAE; Common Terminology Criteria for Adverse Events.

Table S4. Clinical data of case 4

a) age (years old), sex, diagnosis (sites of target lesion), height (cm), body weight (kg) and body surface area (m^2^)

b) change of volume of the target lesion and evaluation of lesions

c) secondary assessments; pleural effusion, ascites, blood coagulation test (platelet counts, fibrinogen and D-dimer), bleeding scales (the World Health Organization-Bleeding Scale), pain scales (visual analog scales), Functional Assessment of Cancer Therapy-General (FACT-G) (>25 years old)) and performance status (PS) (Karnofsky Performance Status (>10 years old), Lansky play-performance scale (for patients <10 years old), Quality of Life (QOL) scores (PedsQL™ 4.0 Generic Core Scales (< 25 years old), and change of other clinical symptoms,.

d) adverse events; events and grade (the Common Terminology Criteria for Adverse Events V4.0) divided into periods (after 0-12 weeks, 13-24 weeks, 25-52 weeks) (underline: causal relationship cannot be ruled out)

| a) 8 months old, female, Klippel-Trenanay-Weber syndrome (right leg, subcutaneous), 65cm, 10.2kg, 0.43m^2^ | | | | |
| --- | --- | --- | --- | --- |
| b) | | | | |
|  | Pre-treatment | After 12 weeks | After 24 weeks | After 52 weeks |
| Change of volume of the target lesion |  | -32% | -22.4% | -20.5% |
| Evaluation of lesions |  | Partial response | Partial response | Partial response |
| c) | | | | |
|  | Pre-treatment | After 12 weeks | After 24 weeks | After 52 weeks |
| Pleural effusion | - | - | - | - |
| Ascites | - | - | - | - |
| Platelet counts (/ul) | 30.9 | 44.3 | 54.5 | 37.3 |
| Fibrinogen (mg/dl) | 279 | 398 | 396 | 281 |
| D-dimer (ug/ml) | 4.4 | 2.6 | 4.5 | 1.8 |
| Bleeding scale | Grade1 | None | None | None |
| Pain scale | - | - | - | - |
| PS (Lansky) | 70 | 90 | 90 | 90 |
| QOL (Peds-QL) | 52.8 | 52.1 | 59.7 | 61.1 |
| Change of other clinical symptoms |  | Unchanged | The lesion became softer | The lesion became softer |
| d) | | | | |
|  | 0-12 weeks | 13-24 weeks | 25-52 weeks |  |
| Events (CTCAE grade) | Fever (1), fever (2), and diaper rash (2) | Fever (2), diaper rash (2), fever (2), and constipation (2) | Fever (2), fever (2), RS virus infection (3), fever (2), and middle otitis (2) |  |

PS; performance status, QOL; Quality of Life, N.A.; not analyzed. -; no data, CTCAE; Common Terminology Criteria for Adverse Events.

Table S5. Clinical data of case 5

a) age (years old), sex, diagnosis (sites of target lesion), height (cm), body weight (kg) and body surface area (m^2^)

b) change of volume of the target lesion and evaluation of lesions

c) secondary assessments; pleural effusion, ascites, blood coagulation test (platelet counts, fibrinogen and D-dimer), bleeding scales (the World Health Organization-Bleeding Scale), pain scales (visual analog scales), Functional Assessment of Cancer Therapy-General (FACT-G) (>25 years old)) and performance status (PS) (Karnofsky Performance Status (>10 years old), Lansky play-performance scale (for patients <10 years old), Quality of Life (QOL) scores (PedsQL™ 4.0 Generic Core Scales (< 25 years old), and change of other clinical symptoms,.

d) adverse events; events and grade (the Common Terminology Criteria for Adverse Events V4.0) divided into periods (after 0-12 weeks, 13-24 weeks, 25-52 weeks) (underline: causal relationship cannot be ruled out)

| a) 71 years old, female, cystic lymphatic malformation (axillary fossa), 154cm, 67.0kg, 1.69m^2^ | | | | |  |
| --- | --- | --- | --- | --- | --- |
| b) | | | | |  |
|  | Pre-treatment | After 12 weeks | After 24 weeks | After 52 weeks |  |
| Change of volume of the target lesion |  | -29.0% | 13.2% | 9.8% |  |
| Evaluation of lesions |  | Partial response | Progressive disease | Progressive disease |  |
| c) | | | | |  |
|  | Pre-treatment | After 12 weeks | After 24 weeks | After 52 weeks |  |
| Pleural effusion | - | - | - | - | |
| Ascites | - | - | - | - | |
| Platelet counts (/ul) | 24.8 | 24.1 | 24.5 | 20.5 | |
| Fibrinogen (mg/dl) | 348 | 426 | 342 | 372 | |
| D-dimer (ug/ml) | 0.5 | 2.4 | 1.2 | 1.2 | |
| Bleeding scale | None | None | None | None | |
| Pain scale | 0 | 3 | 1 | 1 | |
| PS (Karnofsky) | 80 | 90 | 90 | 90 | |
| QOL (FACT-G) | 81.3 | 83.3 | 78.0 | 76.0 | |
| Change of other clinical symptoms |  | Decrease of lymphorrhoea | Lymph fluid had accumulated inside the cyst. | Unchanged | |
| d) | | | | | |
|  | 0-12 weeks | 13-24 weeks | 25-52 weeks |  | |
| Events (CTCAE grade) | None | Acne (2), raising ofγ-glutamyl transferase (3), and stomatitis (1) | Fever (1), abdominal pain upper (1), raising of alanine aminotransferase (3) and lymphocytopenia (3) |  | |

PS; performance status, QOL; Quality of Life, FACT-G; Functional Assessment of Cancer Therapy-General, N.A.; not analyzed. -; no data, CTCAE; Common Terminology Criteria for Adverse Events.

Table S6. Clinical data of case 6

a) age (years old), sex, diagnosis (sites of target lesion), height (cm), body weight (kg) and body surface area (m^2^)

b) change of volume of the target lesion and evaluation of lesions

c) secondary assessments; pleural effusion, ascites, blood coagulation test (platelet counts, fibrinogen and D-dimer), bleeding scales (the World Health Organization-Bleeding Scale), pain scales (visual analog scales), Functional Assessment of Cancer Therapy-General (FACT-G) (>25 years old)) and performance status (PS) (Karnofsky Performance Status (>10 years old), Lansky play-performance scale (for patients <10 years old), Quality of Life (QOL) scores (PedsQL™ 4.0 Generic Core Scales (< 25 years old), and change of other clinical symptoms,.

d) adverse events; events and grade (the Common Terminology Criteria for Adverse Events V4.0) divided into periods (after 0-12 weeks, 13-24 weeks, 25-52 weeks) (underline: causal relationship cannot be ruled out)

| a) 4 years old, male, Klippel-Trenanay-syndrome (left leg), 88cm, 12.6kg, 0.55m^2^ | | | | |
| --- | --- | --- | --- | --- |
| b) | | | | |
|  | Pre-treatment | After 12 weeks | After 24 weeks | After 52 weeks |
| Change of volume of the target lesion |  | -16.0% | -13.9% | -11.8% |
| Evaluation of lesions |  | Stable disease | Stable disease | Stable disease |
| c) | | | | |
|  | Pre-treatment | After 12 weeks | After 24 weeks | After 52 weeks |
| Pleural effusion | - | - | - | - |
| Ascites | - | - | - | - |
| Platelet counts (/ul) | 12 | 16.2 | 19.2 | 16.4 |
| Fibrinogen (mg/dl) | 83 | 91 | 78 | 58 |
| D-dimer (ug/ml) | 26.9 | 44 | 16.6 | 33.1 |
| Bleeding scale | Grade2 | Grade2 | Grade2 | Grade2 |
| Pain scale | 2 | 5 | 78 | 2 |
| PS (Lansky) | 90 | 90 | 90 | 90 |
| QOL (Peds-QL) | 65.5 | 53.6 | 41.7 | 60.7 |
| Change of other clinical symptoms |  | Unchanged | Unchanged | Unchanged |
| d) | | | | |
|  | 0-12 weeks | 13-24 weeks | 25-52 weeks |  |
| Events (CTCAE grade) | Fever (2), upper respiratory inflammation (2), and　pharyngitis (2) | Stomatitis (2) and proteinuria (3) | Loss of consciousness (2), loss of consciousness (2), RS virus infection (2), middle otitis (2), cough (1), and pain (1) |  |

PS; performance status, QOL; Quality of Life, N.A.; not analyzed. -; no data, CTCAE; Common Terminology Criteria for Adverse Events.

Table S7. Clinical data of case 7

a) age (years old), sex, diagnosis (sites of target lesion), height (cm), body weight (kg) and body surface area (m^2^)

b) change of volume of the target lesion and evaluation of lesions

c) secondary assessments; pleural effusion, ascites, blood coagulation test (platelet counts, fibrinogen and D-dimer), bleeding scales (the World Health Organization-Bleeding Scale), pain scales (visual analog scales), Functional Assessment of Cancer Therapy-General (FACT-G) (>25 years old)) and performance status (PS) (Karnofsky Performance Status (>10 years old), Lansky play-performance scale (for patients <10 years old), Quality of Life (QOL) scores (PedsQL™ 4.0 Generic Core Scales (< 25 years old), and change of other clinical symptoms,.

d) adverse events; events and grade (the Common Terminology Criteria for Adverse Events V4.0) divided into periods (after 0-12 weeks, 13-24 weeks, 25-52 weeks) (underline: causal relationship cannot be ruled out)

| a) 3 years old, female, cystic lymphatic malformation (cervical), 95cm, 14.2kg, 0.61m^2^ | | | | |  |
| --- | --- | --- | --- | --- | --- |
| b) | | | | |  |
|  | Pre-treatment | After 12 weeks | After 24 weeks | After 52 weeks |  |
| Change of volume of the target lesion |  | -21.4% | -20.7% | -37.8% |  |
| Evaluation of lesions |  | Partial response | Partial response | Partial response |  |
| c) | | | | |  |
|  | Pre-treatment | After 12 weeks | After 24 weeks | After 52 weeks |  |
| Pleural effusion | - | - | - | - |  |
| Ascites | - | - | - | - |  |
| Platelet counts (/ul) | 38 | 27.3 | 35.6 | 30 |  |
| Fibrinogen (mg/dl) | 254 | 184 | 243 | 335 |  |
| D-dimer (ug/ml) | <0.5 | <0.5 | <0.5 | 0.5 | |
| Bleeding scale | None | None | None | None | |
| Pain scale | - | - | - | - | |
| PS (Lansky) | 100 | 100 | 100 | 100 | |
| QOL (Peds-QL) | 93.1 | 90.5 | 90.5 | 82.1 | |
| Skin lesion | - | - | - | - | |
| Change of other clinical symptoms |  | - | - | - | |
| d) | | | | |  |
|  | 0-12 weeks | 13-24 weeks | 25-52 weeks |  |  |
| Events (CTCAE grade) | - | Acne (2) and upper respiratory inflammation (2) | Stomatitis (1), conjunctivitis (2) and upper respiratory inflammation (2) |  |  |

PS; performance status, QOL; Quality of Life, N.A.; not analyzed. -; no data, CTCAE; Common Terminology Criteria for Adverse Events.

Table S8. Clinical data of case 8

a) age (years old), sex, diagnosis (sites of target lesion), height (cm), body weight (kg) and body surface area (m^2^)

b) change of volume of the target lesion and evaluation of lesions

c) secondary assessments; pleural effusion, ascites, blood coagulation test (platelet counts, fibrinogen and D-dimer), bleeding scales (the World Health Organization-Bleeding Scale), pain scales (visual analog scales), Functional Assessment of Cancer Therapy-General (FACT-G) (>25 years old)) and performance status (PS) (Karnofsky Performance Status (>10 years old), Lansky play-performance scale (for patients <10 years old), Quality of Life (QOL) scores (PedsQL™ 4.0 Generic Core Scales (< 25 years old), and change of other clinical symptoms,.

d) adverse events; events and grade (the Common Terminology Criteria for Adverse Events V4.0) divided into periods (after 0-12 weeks, 13-24 weeks, 25-52 weeks) (underline: causal relationship cannot be ruled out)

| a) 16 years old, male, venous malformation (from left shoulder to forearm), 170cm, 52.4kg, 1.57m^2^ | | | | |  |
| --- | --- | --- | --- | --- | --- |
| b) | | | | |  |
|  | Pre-treatment | After 12 weeks | After 24 weeks | After 52 weeks |  |
| Change of volume of the target lesion |  | -34.7% | -43.1% | -26.7% |  |
| Evaluation of lesions |  | Partial response | Partial response | Partial response |  |
| c) | | | | |  |
|  | Pre-treatment | After 12 weeks | After 24 weeks | After 52 weeks |  |
| Pleural effusion | - | - | - | - |  |
| Ascites | - | - | - | - | |
| Platelet counts (/ul) | 24.5 | 25.1 | 21.6 | 24.9 | |
| Fibrinogen (mg/dl) | 163 | 239 | 230 | 204 | |
| D-dimer (ug/ml) | 1.4 | 1.9 | 0.9 | 0.6 | |
| Bleeding scale | None | None | None | None | |
| Pain scale | 68 | 72 | 83 | 92 | |
| PS (Karnofsky) | 60 | 60 | 60 | 60 | |
| QOL (Peds-QL) | 43.5 | 51.1 | 64.1 | 52.2 | |
| Change of other clinical symptoms |  | Unchanged | Unchanged | Unchanged |  |
| d) | | | | |  |
|  | 0-12 weeks | 13-24 weeks | 25-52 weeks |  |  |
| Events (CTCAE grade) | Headache (1), fatigue, (1), fever (1), stomatitis (2), upper respiratory inflammation (2) and nausea (2) | Diarrhea (1) | Diarrhea (2), insomnia (2) and stomatitis (1) |  |  |

PS; performance status, QOL; Quality of Life, N.A.; not analyzed. -; no data, CTCAE; Common Terminology Criteria for Adverse Events.

Table S9. Clinical data of case 9

a) age (years old), sex, diagnosis (sites of target lesion), height (cm), body weight (kg) and body surface area (m^2^)

b) change of volume of the target lesion and evaluation of lesions

c) secondary assessments; pleural effusion, ascites, blood coagulation test (platelet counts, fibrinogen and D-dimer), bleeding scales (the World Health Organization-Bleeding Scale), pain scales (visual analog scales), Functional Assessment of Cancer Therapy-General (FACT-G) (>25 years old)) and performance status (PS) (Karnofsky Performance Status (>10 years old), Lansky play-performance scale (for patients <10 years old), Quality of Life (QOL) scores (PedsQL™ 4.0 Generic Core Scales (< 25 years old), and change of other clinical symptoms,.

d) adverse events; events and grade (the Common Terminology Criteria for Adverse Events V4.0) divided into periods (after 0-12 weeks, 13-24 weeks, 25-52 weeks) (underline: causal relationship cannot be ruled out)

| a) 14 years old, male, blue rubber bleb nevus syndrome (knee), 149cm, 32.6kg, 1.16m^2^ | | | | |  |
| --- | --- | --- | --- | --- | --- |
| b) | | | | |  |
|  | Pre-treatment | After 12 weeks | After 24 weeks | After 52 weeks |  |
| Change of volume of the target lesion |  | 50.3% | -4.5% | 73.3% |  |
| Evaluation of lesions |  | Progressive disease | Stable disease | Progressive disease |  |
| c) | | | | |  |
|  | Pre-treatment | After 12 weeks | After 24 weeks | After 52 weeks |  |
| Pleural effusion | - | - | - | - |  |
| Ascites | - | - | - | - |  |
| Platelet counts (/ul) | 21.8 | 25.2 | 29.1 | 30 |  |
| Fibrinogen (mg/dl) | 167 | 311 | 204 | 211 |  |
| D-dimer (ug/ml) | 0.7 | 0.8 | <0.5 | 1.4 |  |
| Bleeding scale | - | - | - | - |  |
| Pain scale | 0 | 18 | 58 | 1 |  |
| PS (Karnofsky) | 80 | 80 | 80 | 70 |  |
| QOL (Peds-QL) | 18.5 | 50.0 | 68.5 | 53.3 | |
| Change of other clinical symptoms |  | Improvement of anemia (Hb 10.7g/dL at pre-treatment→ 13.1g/dL) | Unchanged | Unchanged |  |
| d) | | | | |  |
|  | 0-12 weeks | 13-24 weeks | 25-52 weeks |  |  |
| Events (CTCAE grade) | head discomfort (1), abdominal pain (1), neutropenia (2), fatigue (1), stomatitis (1), headache (1), fever (1), fever (1) and fever (1) | Stomatitis (1), stomatitis (1), and stomatitis (1) | Fever (1), fever (1), fever (1), fever (2), pharyngitis (2), fever (2), lower respiratory tract infection (2), lower gastrointestinal bleeding (3) and neutropenia (2) |  |  |

PS; performance status, QOL; Quality of Life, N.A.; not analyzed. -; no data, CTCAE; Common Terminology Criteria for Adverse Events.

Table S10. Clinical data of case 10

a) age (years old), sex, diagnosis (sites of target lesion), height (cm), body weight (kg) and body surface area (m^2^)

b) change of volume of the target lesion and evaluation of lesions

c) secondary assessments; pleural effusion, ascites, blood coagulation test (platelet counts, fibrinogen and D-dimer), bleeding scales (the World Health Organization-Bleeding Scale), pain scales (visual analog scales), Functional Assessment of Cancer Therapy-General (FACT-G) (>25 years old)) and performance status (PS) (Karnofsky Performance Status (>10 years old), Lansky play-performance scale (for patients <10 years old), Quality of Life (QOL) scores (PedsQL™ 4.0 Generic Core Scales (< 25 years old), and change of other clinical symptoms,.

d) adverse events; events and grade (the Common Terminology Criteria for Adverse Events V4.0) divided into periods (after 0-12 weeks, 13-24 weeks, 25-52 weeks) (underline: causal relationship cannot be ruled out)

| a) 6 years old, female, Generalized lymphatic anomaly (right thoracoabdominal), 111cm, 19.7kg, 0.78m^2^ | | | | |
| --- | --- | --- | --- | --- |
| b) | | | | |
|  | Pre-treatment | After 12 weeks | After 24 weeks | After 52 weeks |
| Change of volume of the target lesion |  | -2.5% | -1.5% | 3.1% |
| Evaluation of lesions |  | Stable disease | Stable disease | Stable disease |
| c) | | | | |
|  | Pre-treatment | After 12 weeks | After 24 weeks | After 52 weeks |
| Pleural effusion | - | - | - | - |
| Ascites | - | - | - | - |
| Platelet counts (/ul) | 22.6 | 31 | 29.5 | 34 |
| Fibrinogen (mg/dl) | 170 | 190 | 234 | 245 |
| D-dimer (ug/ml) | 2.7 | 1.4 | 1 | 1.6 |
| Bleeding scale | None | None | None | None |
| Pain scale | 0 | 0 | 0 | 0 |
| PS (Lansky) | 90 | 90 | 90 | 90 |
| QOL (Peds-QL) | 80.4 | 93.5 | 97.8 | 100.0 |
| Change of other clinical symptoms |  | Unchanged | Unchanged | Unchanged |
| d) | | | | |
|  | 0-12 weeks | 13-24 weeks | 25-52 weeks |  |
| Events (CTCAE grade) | Stomatitis （1）, abdominal pain（1）, fever （1）, stomatitis （1）, hyperglyceridemia (2) stomatitis （1）,  and neutropenia (3) | Stomatitis (1) | Stomatitis (1), stomatitis (1), and lower respiratory tract infection (2) |  |

PS; performance status, QOL; Quality of Life, N.A.; not analyzed. -; no data, CTCAE; Common Terminology Criteria for Adverse Events.

Table S11. Clinical data of case 11

a) age (years old), sex, diagnosis (sites of target lesion), height (cm), body weight (kg) and body surface area (m^2^)

b) change of volume of the target lesion and evaluation of lesions

c) secondary assessments; pleural effusion, ascites, blood coagulation test (platelet counts, fibrinogen and D-dimer), bleeding scales (the World Health Organization-Bleeding Scale), pain scales (visual analog scales), Functional Assessment of Cancer Therapy-General (FACT-G) (>25 years old)) and performance status (PS) (Karnofsky Performance Status (>10 years old), Lansky play-performance scale (for patients <10 years old), Quality of Life (QOL) scores (PedsQL™ 4.0 Generic Core Scales (< 25 years old), and change of other clinical symptoms,.

d) adverse events; events and grade (the Common Terminology Criteria for Adverse Events V4.0) divided into periods (after 0-12 weeks, 13-24 weeks, 25-52 weeks) (underline: causal relationship cannot be ruled out)

| a) 6 years old, female, Klippel-Trenanay-Weber syndrome (right leg), 135cm, 31.0kg, 1.08m^2^ | | | | |  |
| --- | --- | --- | --- | --- | --- |
| b) | | | | |  |
|  | Pre-treatment | After 12 weeks | After 24 weeks | After 52 weeks |  |
| Change of volume of the target lesion |  | 5.3% | -16.1% | -44.1% |  |
| Evaluation of lesions |  | Stable disease | Stable disease | Partial response |  |
| c) | | | | |  |
|  | Pre-treatment | After 12 weeks | After 24 weeks | After 52 weeks | |
| Pleural effusion | - | - | - | - | |
| Ascites | - | - | - | - | |
| Platelet counts (/ul) | 20.1 | 24.6 | 25.5 | 28.2 | |
| Fibrinogen (mg/dl) | 217 | 245 | 259 | 258 | |
| D-dimer (ug/ml) | 3.4 | 2.4 | 3.6 | 1.7 | |
| Bleeding scale | None | None | None | None | |
| Pain scale | 0 | 0 | 0 | 0 | |
| PS (Lansky) | 100 | 100 | 100 | 100 | |
| QOL (Peds-QL) | 100.0 | 100.0 | 100.0 | 100.0 | |
| Change of other clinical symptoms |  | Unchanged | Dressing and undressing became smoother. | Unchanged | |
| d) | | | | |  |
|  | 0-12 weeks | 13-24 weeks | 25-52 weeks |  |  |
| Events (CTCAE grade) | Stomatitis （1）, stomatitis （1）, stomatitis （1）, upper respiratory inflammation (2), stomatitis （1）, and cellulitis(3) | Stomatitis (1), nosebleed (1), stomatitis (1), and neutropenia (3) | Stomatitis (1), cellulitis (3), stomatitis (1), stomatitis (1), cellulitis (3), cystitis (1), dermatitis (2), cellulitis (3), and cellulitis (3) |  |  |

PS; performance status, QOL; Quality of Life, N.A.; not analyzed. -; no data, CTCAE; Common Terminology Criteria for Adverse Events.

Table S12. Clinical data of case 13

a) age (years old), sex, diagnosis (sites of target lesion), height (cm), body weight (kg) and body surface area (m^2^)

b) change of volume of the target lesion and evaluation of lesions

c) secondary assessments; pleural effusion, ascites, blood coagulation test (platelet counts, fibrinogen and D-dimer), bleeding scales (the World Health Organization-Bleeding Scale), pain scales (visual analog scales), Functional Assessment of Cancer Therapy-General (FACT-G) (>25 years old)) and performance status (PS) (Karnofsky Performance Status (>10 years old), Lansky play-performance scale (for patients <10 years old), Quality of Life (QOL) scores (PedsQL™ 4.0 Generic Core Scales (< 25 years old), and change of other clinical symptoms,.

d) adverse events; events and grade (the Common Terminology Criteria for Adverse Events V4.0) divided into periods (after 0-12 weeks, 13-24 weeks, 25-52 weeks) (underline: causal relationship cannot be ruled out)

| a) 1 years old, female, Kaposiform hemangioendothelioma (left shoulder), 71cm, 8.4kg, 0.41m^2^ | | | | |  |
| --- | --- | --- | --- | --- | --- |
| b) | | | | |  |
|  | Pre-treatment | After 12 weeks | After 24 weeks | After 52 weeks |  |
| Change of volume of the target lesion |  | -33.1% | -64.0% | -74.7% |  |
| Evaluation of lesions |  | Partial response | Partial response | Partial response |  |
| c) | | | | |  |
|  | Pre-treatment | After 12 weeks | After 24 weeks | After 52 weeks | |
| Pleural effusion | - | - | - | - | |
| Ascites | - | - | - | - | |
| Platelet counts (/ul) | 17 | 33.8 | 39.4 | 48.6 | |
| Fibrinogen (mg/dl) | 192 | 299 | 354 | 287 | |
| D-dimer (ug/ml) | 23.3 | 1.6 | <0.5 | <0.5 | |
| Bleeding scale | None | None | None | None | |
| Pain scale | - | - | - | - | |
| PS (Lansky) | 70 | 90 | 90 | 90 | |
| QOL (Peds-QL) | 82.2 | 87.8 | 83.9 | 95.6 | |
| Change of other clinical symptoms |  | Improvement of Kasabach-Merritt phenomenon | Normalization of coagulopathy | Normalization of shoulder joint range of motion |  |
| d) | | | | |  |
|  | 0-12 weeks | 13-24 weeks | 25-52 weeks |  |  |
| Events (CTCAE grade) | Fever (1), stomatitis (2), diarrhea (2), neutropenia (3), stomatitis (2), rhinorrhea (2) and upper respiratory infection (2) | Stomatitis (1) | Fever (2) and nasopharyngitis (2) |  |  |

PS; performance status, QOL; Quality of Life, N.A.; not analyzed. -; no data, CTCAE; Common Terminology Criteria for Adverse Events.

Table S13. Clinical data of case 14

a) age (years old), sex, diagnosis (sites of target lesion), height (cm), body weight (kg) and body surface area (m^2^)

b) change of volume of the target lesion and evaluation of lesions

c) secondary assessments; pleural effusion, ascites, blood coagulation test (platelet counts, fibrinogen and D-dimer), bleeding scales (the World Health Organization-Bleeding Scale), pain scales (visual analog scales), Functional Assessment of Cancer Therapy-General (FACT-G) (>25 years old)) and performance status (PS) (Karnofsky Performance Status (>10 years old), Lansky play-performance scale (for patients <10 years old), Quality of Life (QOL) scores (PedsQL™ 4.0 Generic Core Scales (< 25 years old), and change of other clinical symptoms,.

d) adverse events; events and grade (the Common Terminology Criteria for Adverse Events V4.0) divided into periods (after 0-12 weeks, 13-24 weeks, 25-52 weeks) (underline: causal relationship cannot be ruled out)

| a) 1 years old, female, cystic lymphatic malformation (cervical lesion), 75cm, 9.4kg, 0.44m^2^ | | | | |  |
| --- | --- | --- | --- | --- | --- |
| b) | | | | |  |
|  | Pre-treatment | After 12 weeks | After 24 weeks | After 52 weeks |  |
| Change of volume of the target lesion |  | -32.4% | 10.2% | - (The test could not be conducted due to fever caused by respiratory tract infection) |  |
| Evaluation of lesions |  | Partial response | Progressive disease | N.A. |  |
| c) | | | | |  |
|  | Pre-treatment | After 12 weeks | After 24 weeks | After 52 weeks |  |
| Pleural effusion | - | - | - | - |  |
| Ascites | - | - | - | - |  |
| Platelet counts (/ul) | 46 | 41.8 | 44.4 | 34.7 |  |
| Fibrinogen (mg/dl) | 295 | 395 | 475 | 516 |  |
| D-dimer (ug/ml) | <0.5 | <0.5 | 0.6 | <0.5 |  |
| Bleeding scale | Grade 1^#^ | None | Grade 1^#^ | None |  |
| Pain scale | - | - | - | - |  |
| PS (Lansky) | 60 | 80 | 90 | 90 | |
| QOL (Peds-QL) | 81.1 | 78.3 | 81.7 | 94.4 | |
| Change of other clinical symptoms |  | Improvement of swelling and bleeding of the tongue | Bleeding in the affected area | Improvement of swelling and bleeding of the tongue | |
| d) | | | | |  |
|  | 0-12 weeks | 13-24 weeks | 25-52 weeks |  |  |
| Events (CTCAE grade) | Dry skin (2), fever (1), diarrhea (2), fever (1), and upper respiratory infection (3) | Bronchitis (3) | RS virus infection (3), fever (2) and respiratory tract infection (1) |  |  |

# Intralesional bleeding

PS; performance status, QOL; Quality of Life, N.A.; not analyzed. -; no data, CTCAE; Common Terminology Criteria for Adverse Events.
